# An improved Sinh Cosh optimizer for optimizing energy management system in nano-grids

**DOI:** 10.1038/s41598-025-16955-w

**Published:** 2025-09-12

**Authors:** Asmaa H. Rabie, Sally Elghamrawy, Aboul Ella Hassanien

**Affiliations:** 1 Computers and Control Systems Engineering Department, Faculty of Engineering, Mansoura University, Mansoura, Egypt; 2 Computer Engineering Department, MISR Higher Institute for Engineering and Technology, Mansoura, Egypt; 3 Faculty of Computers and Artificial Intelligence, Cairo University, Cairo, Egypt; 4 Scientific Research School of Egypt (SRSEG), Cairo, Egypt

**Keywords:** Nano-grid, Energy management systems, Renewable energy, Meta-heuristic algorithms, Improved sinh cosh optimizer, Electrical and electronic engineering, Energy grids and networks, Renewable energy

## Abstract

The increasing integration of renewable energy sources in Nano-grids has created a need for efficient energy management systems to optimize energy usage and minimize operational costs. Traditional optimization algorithms often struggle with balancing the complex trade-offs between different energy sources, such as wind, solar, natural gas generators, and batteries, resulting in suboptimal performance and higher costs. To address this challenge, this paper introduces the Improved Sinh Cosh Optimizer (ISCHO), a novel meta-heuristic algorithm designed to enhance the energy management system in Nano-grids. ISCHO mimics the characteristics of Sinh and Cosh functions to dynamically adjust the balance between exploration and exploitation, enabling more efficient search space exploration and convergence towards optimal solutions. By optimizing key parameters related to energy generation and storage, ISCHO minimizes the total operational cost of the Nano-grid. Simulation results show that ISCHO outperforms traditional methods by achieving a significant reduction in total costs, making it a robust solution for real-time energy management in Nano-grids. According to population sizes equal 500 and 1000, ISCHO gave the best fitness values, means, and standard deviations of 0, which refered to a significant reduction in total operational costs. For instance, at a population size of 500, ISCHO’s fitness value of 0 was significantly lower than the highest fitness value of 23.768 × 10^− 6^ recorded by the Chimp algorithm. Furthermore, ISCHO maintained a competitive execution time (e.g., 3.00862 s for population 500), confirming its practical applicability for real-time energy management in Nano-grids. Additionally, results ensured that ISCHO outperformed other algorithms using five benchmark functions. Hence, ISCHO’s competitive execution time further solidifies its effectiveness for real-time energy management in Nano-grids.

## Introduction

Energy management systems (EMS) in Nano-grids are increasingly recognized as a basis in the transition toward decentralized, sustainable, and resilient power systems. Nano-grids are designed to integrate various renewable energy sources and storage systems, such as solar photovoltaic panels, wind turbines, natural gas generators, and batteries, into compact, autonomous units capable of operating independently or in coordination with the main grid. As global energy systems shift toward low-carbon solutions, Nano-grids offer a promising pathway for enhancing energy self-sufficiency, reducing carbon emissions, and improving local energy reliability.

However, the effective integration and coordination of these heterogeneous energy sources remain a complex challenge. Variations in renewable generation, nonlinear system dynamics, and real-time decision-making requirements require advanced optimization techniques to ensure minimal operational cost, reliable performance, and efficient resource utilization. Traditional energy management methods often fall short in addressing the dynamic and multidimensional nature of Nano-grid systems, making advanced algorithmic solutions not only beneficial but essential. In this context, the development of robust and adaptive optimization strategies is of critical importance.

Meta-heuristic algorithms have been widely applied in energy management optimization due to their ability to handle non-linear and complex problems. Genetic Algorithms (GA)^1^, Particle Swarm Optimization (PSO)^2^, and Differential Evolution (DE)^3^ are among the most prominent methods used in this domain. More recently, nature-inspired algorithms such as the Whale Optimization Algorithm (WOA)^4^, Ant Colony Optimization (ACO)^5^, and Grey Wolf Optimizer (GWO)^6^ have been explored to improve performance in energy management systems. These algorithms offer better convergence properties and adaptability to varying energy input conditions, but their performance is still limited by issues related to exploration and exploitation balance, often resulting in higher computational costs or less accurate results in real-time applications.

To overcome these limitations, the current trend in optimization research focuses on enhancing existing meta-heuristic algorithms through dynamic control strategies, hybridization, and the incorporation of intelligent operators. Techniques such as Fitness-Distance Balance (FDB), adaptive parameter tuning, and local search integration have been proposed to improve exploration-exploitation balance and convergence rates. These strategies are designed to address common challenges like stagnation in local optima and slow convergence, ultimately aiming to develop more efficient and reliable optimizers suitable for complex real-world systems^7–13^.

One of the main components of contemporary optimization research involves the modification and enhancement of existing metaheuristic algorithms to improve their performance across diverse problem domains. In this context, several operators and strategies are frequently employed to refine the exploration and exploitation capabilities of these algorithms. Fitness-Distance Balance (FDB), as a well-known selection technique, is used to direct the search process by taking into account a solution’s fitness as well as its separation from other alternatives. This aims to strike a compromise between diversity preservation and convergence pace. For selecting viable answers for replication or directing the search, a probabilistic method is used by roulette wheel selection improved with FDB principles. Another popular tactic used to increase the algorithm’s adaptability includes dynamic parameters, such as step sizes or inertia weights, that change as the optimization process progresses. Additionally, incorporating local search mechanisms or combining them with other optimization strategies can aid in stepping up the search around areas that the global exploration phase found to be promising. Redesigning exploration operators based on tactics such as the natural survival technique is an example of how changes that are inspired by natural events frequently entail changing the movement or interaction rules of the search agents. By addressing frequent metaheuristic drawbacks, including early convergence and stagnation in local optima, these changes hope to generate optimization algorithms that are more reliable and effective^7–13^.

Among these developments, the Sinh Cosh Optimizer (**SCHO**), introduced by^14^, leverages hyperbolic mathematical functions to manage the exploration–exploitation trade-off in optimization tasks. While SCHO has shown promise in general optimization problems, its application to multi-variable, real-time systems like Nano-grids remains underexplored. Given the increasing penetration of renewables and the critical need for efficient energy scheduling, this gap presents a valuable research opportunity.

This paper introduces the Improved Sinh Cosh Optimizer (ISCHO), a novel meta-heuristic algorithm inspired by the hyperbolic Sinh and Cosh functions, specifically designed for energy management in Nano-grid systems. ISCHO is designed to dynamically adjust its search behavior, offering an enhanced mechanism for balancing global exploration with local exploitation. By optimizing the energy contributions from key resources (wind, solar, natural gas, and battery storage), ISCHO aims to minimize total operational costs while maintaining system reliability and adaptability.

This research is of vital importance, as it provides a scalable, computationally efficient, and accurate approach to managing distributed renewable energy systems. In an era where energy autonomy, sustainability, and cost efficiency are national and global priorities, the contribution of ISCHO to the field of energy optimization is both timely and impactful. The algorithm’s ability to operate under fluctuating energy prices and generation conditions makes it particularly compatible for real-time energy management in next-generation Nano-grids.

The main contributions of this paper are as follows:

- A novel improved optimization algorithm that incorporates the mathematical properties of the Sinh and Cosh functions, namely ISCHO, to balance exploration and exploitation in the search space, improving the convergence rate in energy management systems.
- The proposed ISCHO algorithm is employed to optimize energy allocation among multiple distributed energy sources in a Nano-grid system, namely wind turbines, photovoltaic panels, natural gas generators, and battery storage units. By determining the optimal contribution of each source under varying conditions, ISCHO minimizes the total operational cost while ensuring efficient and reliable energy management.
- The energy management process is outlined as an optimization problem, with the cost function reflecting the energy output from different sources and their associated costs. ISCHO effectively minimizes this cost by dynamically adjusting the energy distribution parameters.
- The proposed algorithm introduces a switching mechanism that adjusts between exploration and exploitation phases based on nonlinear functions. This feature ensures that the algorithm escapes local minima and explores the global search space efficiently.
- Comparative analysis and simulation results demonstrate that ISCHO achieves a lower total cost in Nano-grid management than traditional optimization methods, proving its superior performance in real-time scenarios.

The remainder of the paper is organized as follows. Section 2 reviews the related work in the field. Section 3 introduces the proposed Improved Sinh Cosh Optimizer (ISCHO) for energy management. Section 4 presents the simulation setup and discusses the results. Finally, Sect. 5 concludes the study and outlines potential directions for future work.

## Related work

**Fig. 1:**
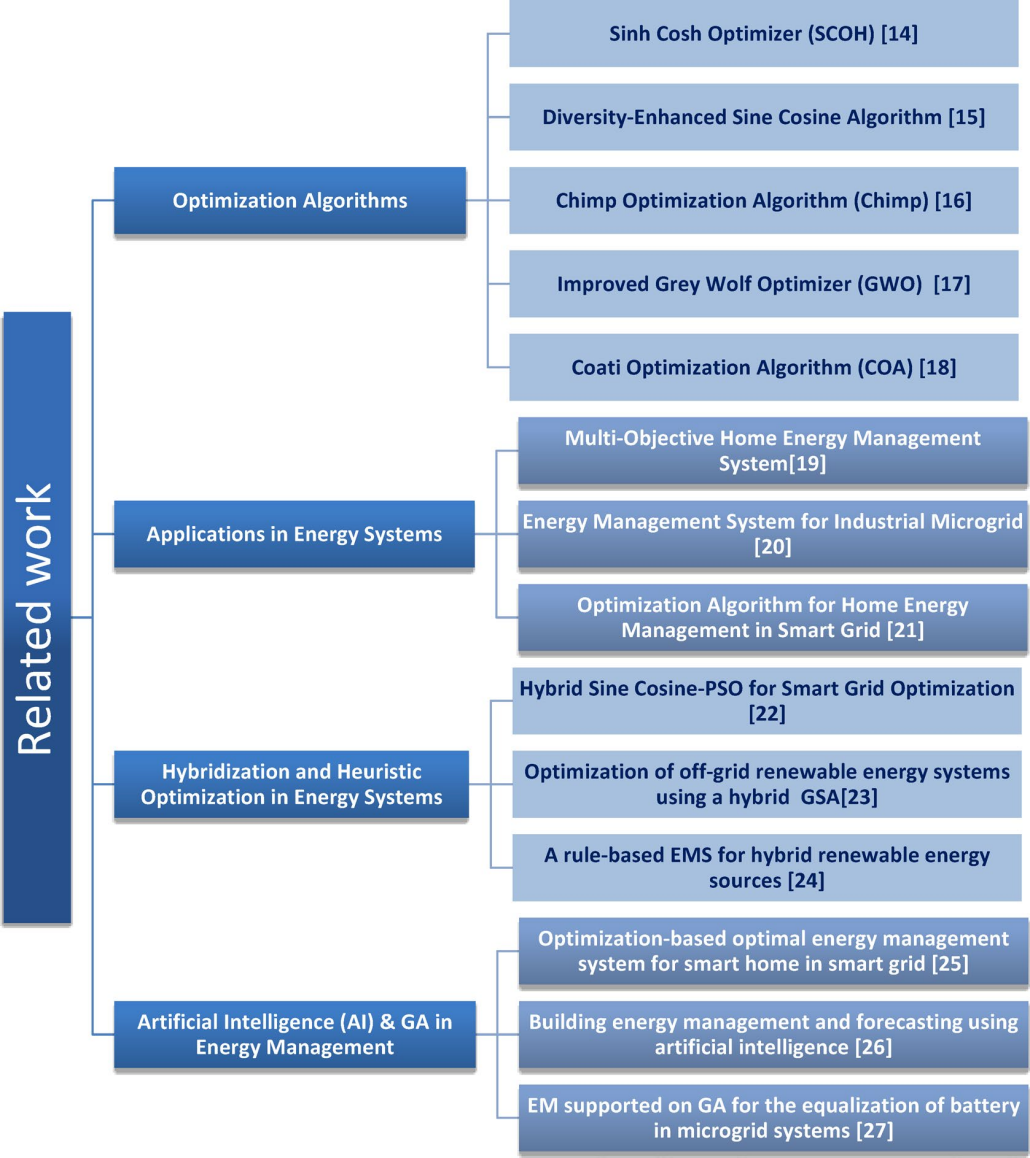
Categorization of the related work in optimization algorithms and energy management systems.

This section surveys the related work related to optimization algorithms and their applications in EMS, with a particular focus on Nano-grid environments. It identifies the main approaches and techniques in the literature, summarizes their strengths and weaknesses, and clarifies the key research gaps addressed by the proposed Improved Sinh Cosh Optimizer (ISCHO). The review categorizes the literature into four thematic areas, as shown in Fig. 1: (i) Optimization algorithms, (ii) Applications in energy systems, (iii) Hybridization and heuristic optimization in energy systems, and (iv) Artificial Intelligence (AI) and Genetic Algorithms (GA) in energy management.

### Optimization algorithms

Metaheuristic algorithms are increasingly applied to complex optimization problems due to their capacity to efficiently navigate vast and non-linear solution spaces. One such approach is the **Sinh Cosh Optimizer (SCHO)**^14^, which utilizes hyperbolic sine and cosine functions to maintain a balance between exploration and exploitation. While SCHO has demonstrated promising results in benchmark optimization problems, its application in dynamic environments like Nano-grid energy systems has not been extensively validated, limiting its practical adoption in such domains.

The **Diversity-Enhanced Sine Cosine Algorithm (SCA)**^15^ improves upon the original SCA by incorporating diversity mechanisms to overcome stagnation, particularly in high-dimensional search spaces. This enhancement improves robustness and the ability to avoid local optima, making it effective for engineering design problems. However, the additional diversity mechanisms may increase computational complexity. Moreover, its utility in domains beyond engineering design, such as energy management systems, remains underexplored. A comparative analysis with other diversity-focused metaheuristics like Differential Evolution (DE) and Genetic Algorithms (GA) would have further clarified its position among optimization strategies. Its real-time applicability for energy management, especially under dynamic and uncertain conditions, also requires further investigation.

The **Chimp Optimization Algorithm (Chimp)**^16^ is another nature-inspired method that mimics the social behavior of chimpanzees, offering superior exploratory capabilities. Its intuitive, behavior-based approach lends itself to multiple domains. However, the algorithm’s performance on constrained or complex problems, such as those found in energy systems, has not been thoroughly evaluated. It lacks comparative analysis with well-established bio-inspired algorithms such as ACO and PSO. Furthermore, there is insufficient discussion on its scalability and convergence behavior in multi-objective or real-time optimization tasks. As a result, its readiness for deployment in large-scale, dynamic environments like energy management systems remains uncertain.

The **Improved Grey Wolf Optimizer (IGWO)**^17^ enhances the standard GWO by offering faster convergence and improved accuracy. These improvements are particularly evident in high-dimensional search spaces, making it well-suited for complex engineering applications. However, the improvements are considered incremental, with limited novelty in algorithmic design. Its applicability in energy systems is not thoroughly tested, and the lack of comprehensive comparisons with other enhanced swarm intelligence algorithms (e.g., improved PSO variants) limits its perceived utility. Moreover, real-world implementation in domains such as IoT-based energy systems or smart microgrids is an area yet to be explored. Similarly, the **Coati Optimization Algorithm (COA)**^18^, inspired by the social behavior of coatis, has shown competitive performance across various benchmark functions. While the algorithm demonstrates general versatility, its effectiveness in managing large-scale or dynamic optimization problems—such as energy management in smart grids or Nano-grids—remains largely theoretical. The scalability, computational efficiency, and comparative performance of COA with established optimization techniques are areas that require deeper empirical analysis. Testing COA in practical, real-world scenarios would be necessary to validate its performance and usability.

### Applications in energy systems

Several studies have explored the integration of optimization algorithms into smart energy systems, especially for energy management in smart homes, microgrids, and industrial settings. For instance, **Multi-Objective Home Energy Management Systems**^19^ combine IoT-enabled architectures with metaheuristic algorithms to balance multiple objectives such as cost, energy efficiency, and user preferences. The inclusion of IoT provides real-time adaptability, which is crucial in dynamic home energy settings. However, scalability issues in large-scale smart grids and a lack of real-world implementation details reduce the generalizability of this approach. Additionally, comparisons with machine learning or reinforcement learning-based energy management solutions would have enriched the analysis. A hybrid approach is presented in **Industrial Microgrid EMS Using Reinforcement Learning and Heuristics**^20^, where reinforcement learning is combined with metaheuristics to optimize energy usage in industrial microgrids. This method shows promise for real-time adaptability in dynamic industrial environments. However, the computational overhead of reinforcement learning in large-scale settings is not fully discussed, and the algorithm’s ability to balance multiple conflicting objectives—such as cost efficiency versus energy savings—is under-analyzed.

The **Artificial Bee Colony (ABC) algorithm** has also been applied in the context of smart grid EMS^21^. ABC is known for its balanced exploration and exploitation capabilities, making it a suitable choice for optimizing dynamic and non-linear environments like smart grids. Nevertheless, the use of an older algorithm without benchmarking it against more recent methods—such as enhanced PSO or hybrid approaches—limits the contribution. A more extensive comparative analysis would have provided deeper insights into the ABC algorithm’s suitability for modern energy management systems.

### Hybridization and heuristic optimization in energy systems

Hybrid algorithms that combine multiple optimization strategies are gaining attention for their ability to improve performance in energy applications. The **Hybrid SCA-PSO for Smart Grid Optimization**^22^ merges the convergence speed of PSO with the diversity properties of SCA. This hybrid algorithm provides a more balanced optimization strategy, making it particularly effective for demand-side energy management in smart grids. While the results are promising, the study does not fully explore the computational complexity introduced by hybridization or its scalability for real-time and multi-objective energy systems. Additionally, the lack of comparison with other hybrid algorithms or practical case studies weakens its impact. Another example is the **Hybrid Golden Search Algorithm (GSA) for Off-grid Renewable Systems**^23^, which optimizes energy generation and storage in renewable-powered systems. The method is specifically designed to enhance the efficiency of energy utilization and resource allocation. Tested across multiple off-grid scenarios, the hybrid GSA demonstrates significant improvements over conventional techniques. However, its computational complexity could hinder its application in real-time systems. The scalability of this algorithm for larger, distributed energy systems is not thoroughly discussed. A different strategy is observed in **Rule-Based EMS with GA for Hybrid Renewables**^24^, which employs a rule-based framework to manage energy generation and consumption in systems using hybrid renewable sources like solar and wind. GA is used to optimize battery charge/discharge cycles, ensuring efficient energy use while maintaining battery health. This real-time decision-making approach offers an efficient way to manage variable energy supply and demand. However, like other GA-based solutions, it may face computational challenges when scaled to more complex or extensive energy systems.

### AI and GA in energy management

Artificial Intelligence (AI) and heuristic optimization methods are being increasingly used in predictive and real-time energy management. In the **Optimization-based EMS for Smart Homes**^25^, an AI-powered optimization strategy is used to balance energy consumption across different sources (renewables, grid, batteries), considering time-of-use pricing and dynamic demand. While the system shows promise in reducing costs and increasing energy efficiency, it does not sufficiently address the variability in user consumption behavior or the complexities of integrating multiple energy sources in real-time. The application of AI in **Building Energy Management and Forecasting**^26^ involves the use of machine learning to predict energy usage patterns. These AI models enhance energy distribution and reduce wastage by learning from historical data. However, their effectiveness depends heavily on the quality and availability of input data. In environments with limited data, the performance of such systems can degrade significantly. Lastly, **GA-based EMS for Battery Equalization in Microgrids**^27^ proposes a GA-driven optimization model to equalize energy distribution across battery storage units in microgrids. The system ensures optimal charge-discharge cycles, contributing to energy efficiency and battery longevity. While the approach is technically sound, its computational demand may hinder real-time performance in larger, more complex systems.

### The research gaps and motivations for ISCHO

Despite the advancements in metaheuristic optimization and AI-driven EMS, the literature reveals several critical gaps: Most algorithms lack dynamic adaptability to real-time changes in energy prices and demand. There is limited integration of multiple energy sources (PV, wind, diesel, battery) into a unified optimization framework. Comparative evaluations using rigorous statistical tools (e.g., t-tests, p-values) are sparse. Scalability and performance under practical Nano-grid constraints are rarely addressed.

To address these challenges, this paper introduces the **Improved Sinh Cosh Optimizer (ISCHO)**, a novel metaheuristic algorithm designed for real-time energy optimization in Nano-grids. ISCHO enhances traditional SCHO through adaptive strategies that dynamically respond to pricing and energy availability. It is validated through statistical comparison and sensitivity analysis, demonstrating superior performance in minimizing operational costs across diverse scenarios.

### Discussion of the related work

This subsection summarizes the related works, it provides a brief summary of various research works focused on optimization algorithms, highlighting their categories, main content, publishers, publication years, and measurable outcomes.

Several existing studies have proposed advanced optimization algorithms for improving energy management and system efficiency across various grid and microgrid configurations. For example, studies^14–18^ introduced novel metaheuristic algorithms such as the Sinh Cosh Optimizer (SCHO), Modified Sine Cosine Algorithm, Chimp Optimization Algorithm (ChOA), Improved Grey Wolf Optimizer (IGWO), and Coati **Optimization** Algorithm (COA), with a focus on enhancing convergence speed, solution quality, and algorithmic diversity. These works generally emphasized parameters like exploration–exploitation balance, solution accuracy, and computational speed, evaluated on benchmark engineering problems. Other works, particularly^25–27^, focused on energy management systems in smart homes or grids, using Internet of Things (IoT) integration and optimization strategies to achieve objectives like cost reduction, load balancing, and energy efficiency. Key parameters analyzed included power consumption profiles, demand response mechanisms, and real-time control of appliances or sources. More recent efforts such as^15–18^ addressed microgrid and off-grid energy system optimization, incorporating hybrid renewable energy sources (wind, solar, batteries) and leveraging algorithms like metaheuristics, reinforcement learning, and hybrid methods (e.g., SCA-PSO). These studies typically assessed system reliability, battery lifespan, operational cost, and sustainability metrics.

Lastly, works like^25–27^ introduced predictive and rule-based optimization frameworks for both smart building energy forecasting and battery energy storage systems. Additionally, the work in^28,29^ introduced a hybrid renewable energy management using different optimization algorithms. These considered forecasting accuracy, energy consumption trends, and optimization of battery charge-discharge cycles as primary parameters. Collectively, these studies inform the design and benchmarking of optimization strategies like the proposed ISCHO, but none have specifically addressed its adaptation to Nano-grid energy distribution involving real-time cost minimization under hybrid generation sources.

**Fig. 2:**
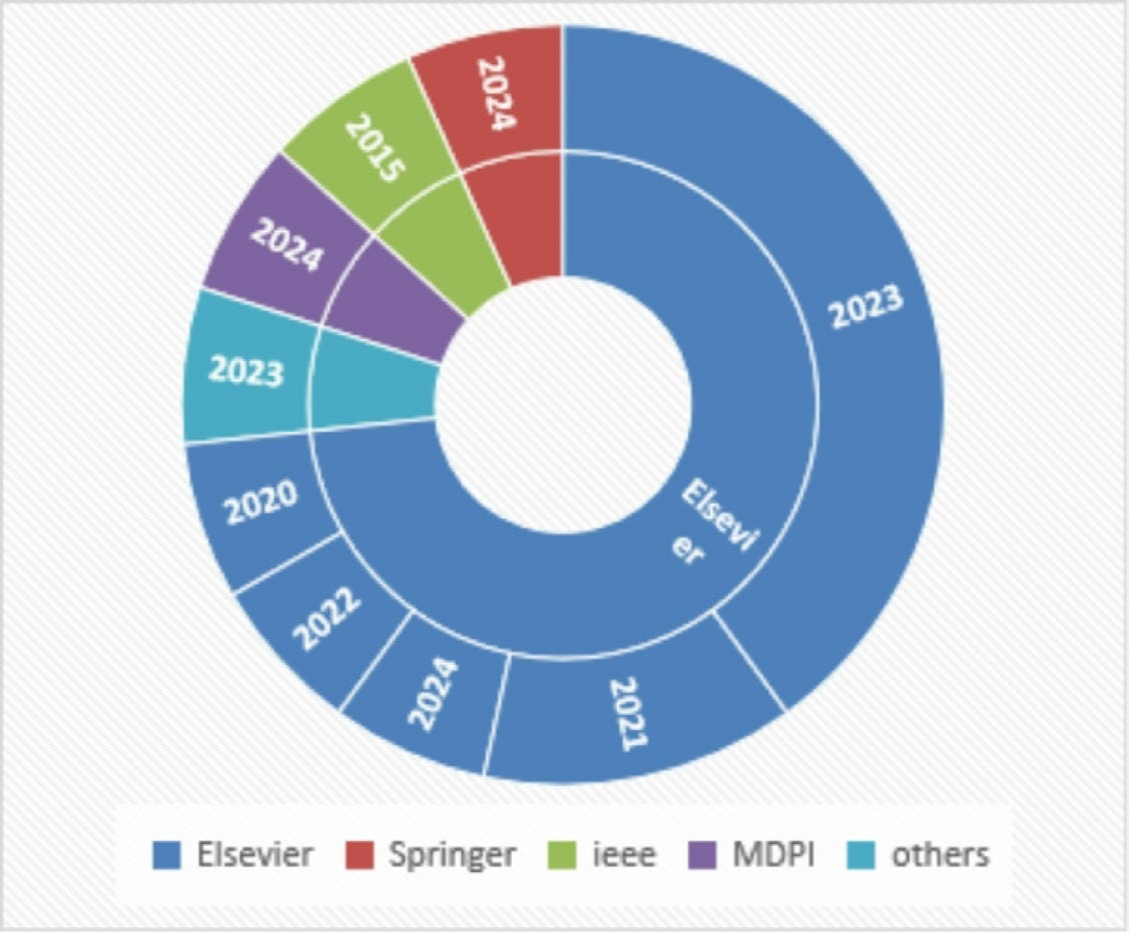
Distribution of publications by publisher and year.

This diagram in Fig. 2 illustrates the distribution of research publications across different publishers (Elsevier, Springer, IEEE, MDPI and others) over several years, from 2015 to 2024.

The largest share of publications is associated with Elsevier, particularly in the years 2020 through 2024. Other publishers, including Springer, IEEE, and MDPI, also show contributions, with their publication years highlighted. The chart provides a clear visual representation of how publication output is distributed among these major publishers over time, emphasizing Elsevier’s dominance in recent years.

The related work highlights various optimization algorithms applied to energy management systems. The related work discussed categorizes optimization algorithms into four main areas, focusing on their applicability in energy systems. Each algorithm brings unique approaches to improving performance in optimization tasks, but many fall short when handling the complexities of Nano-grids, which integrate diverse renewable energy sources like solar, wind, and batteries. Traditional methods often struggle with balancing the trade-offs between different energy sources, resulting in suboptimal performance and higher operational costs. This motivates the development of the ISCHO in this paper, designed to address these challenges by enhancing energy management efficiency in Nano-grids. ISCHO leverages the unique mathematical properties of Sinh and Cosh functions to balance exploration and exploitation, achieving superior results in optimizing energy generation and storage compared to existing algorithms. However, despite the promise of various algorithms, the literature lacks sufficient real-world testing, scalability assessments, and direct comparisons with more established methods. This gap in the existing research further underscores the importance of ISCHO, which not only addresses computational efficiency but also demonstrates practical utility in minimizing operational costs for real-time energy management in Nano-grids.

## The proposed improved Sinh Cosh optimizer (ISCHO) for energy management

**Fig. 3:**
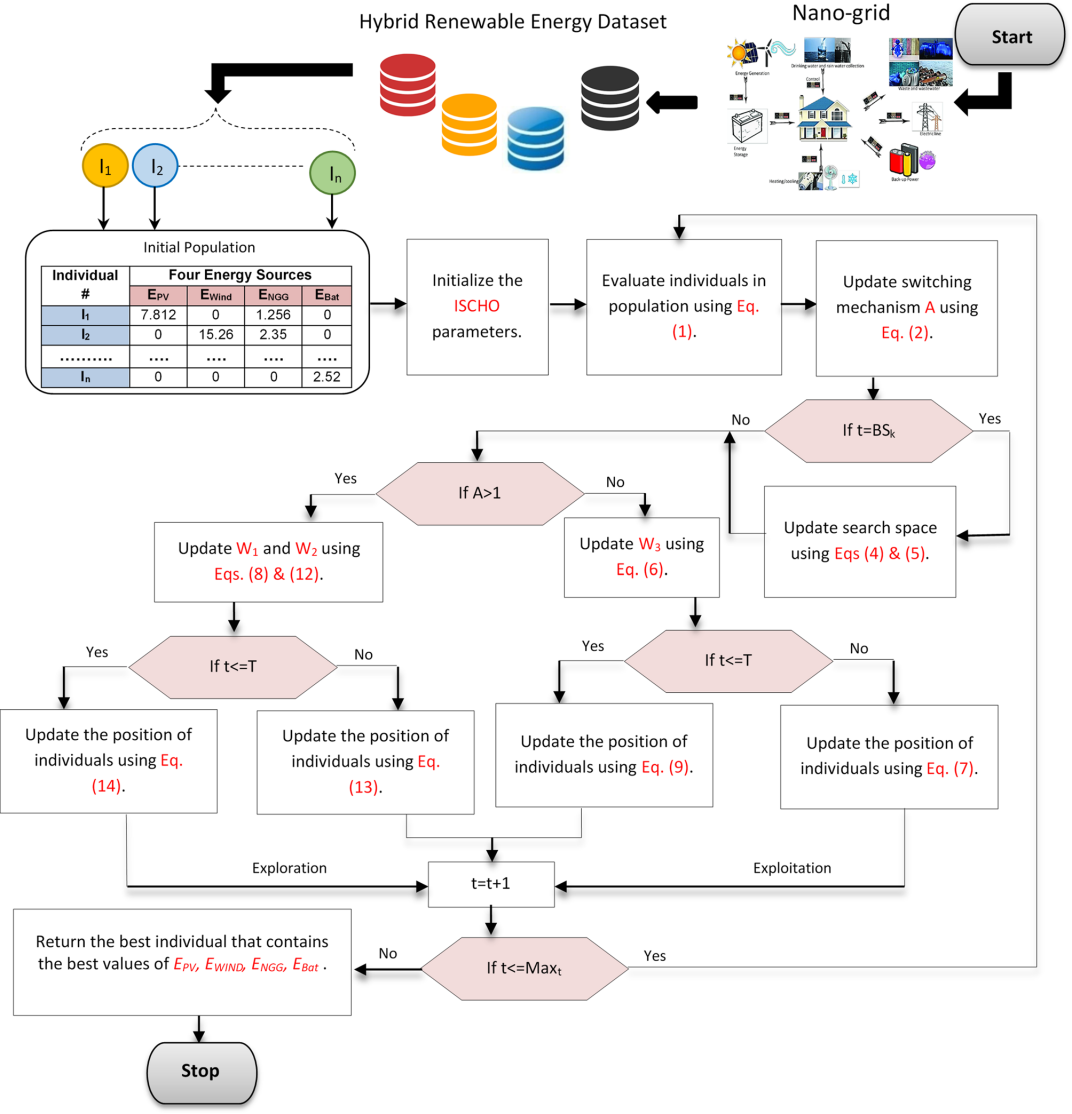
The steps of implementing ISCHO to optimize energy management system.

Nano-grid is a power distribution system that combines the energy storage systems (like batteries) and the hybrid renewable energy sources (like solar and wind) to provide electricity to a specific area^24–27^. The goal is to reduce dependence on the main grid and increase energy self-sufficiency. In this section, the energy management system will be optimized using a new optimization algorithm called the Improved Sinh Cosh Optimizer (ISCHO) to minimize the total cost in the Nano-grid. ISCHO is a new meta-heuristic algorithm that mimics the characteristics of Sinh and Cosh functions^14^. Hence, ISCHO will be used in Nano-grid to optimize the affected parameters in the energy management system, which will reduce the total cost. These parameters (four parameters) are the energy of wind, photovoltaic (PV), Natural Gas Generator (NGG), and Battery (Bat); (E_WIND_, E_PV_, E_NGG_, E_Bat_). The energy management process is represented as an optimization problem where the cost function directly reflects the energy output from each source and its corresponding current price. Dynamically, ISCHO adjusts these energy distribution parameters, and its design incorporates a switching mechanism based on nonlinear functions to balance exploration and exploitation, ensuring efficient search space exploration and convergence to optimal solutions. This minimizes the overall cost by enabling the ISCHO to adjust and offer an optimal energy management strategy even in the event that energy prices fluctuate. Figure 3 outlines the ISCHO implementation procedures, which include position updates, fitness assessment, and population initialization.

The ISCHO, as a new meta-heuristic algorithm, is presented to improve energy management in Nano-grids. The SCHO functions serve as the inspiration for ISCHO, which uses their mathematical characteristics to negotiate the challenges of cost reduction and energy distribution. It can overcome the limitations of conventional optimization algorithms, which have trouble weighing the trade-offs between different energy sources, including batteries, solar, wind, and natural gas. For achieving effective search space exploration and convergence to optimal solutions, ISCHO’s design enables a dynamic adjustment between exploration and exploitation, where exploration refers to searching widely for prospective solutions and exploitation refers to refining promising answers. By optimizing energy generation and storage characteristics, this algorithm improves energy management and lowers the overall operating expenses of Nano-grids. By offering improved methods for updating individual locations, ISCHO may therefore address this issue and enable a more flexible search with a better balance between discovering new areas and taking use of promising ones. ISCHO’s improvements seek to improve convergence speed and yield higher-quality solutions by resolving the inadequacies of SCHO’s position update, especially for complex optimization issues like energy management systems.

According to Fig. 3, ISCHO begins with a population including ‘*n’* individuals. Each individual contains four positions (parameters) with initial random values: *I*_*j*_*={E*_*PV*_, *E*_*WIND*_, *E*_*NGG*_, *E*_*Bat*_*}*. Next, a fitness function that represents the total cost in a Nano-grid will be used to test every individual in the population. This fitness (cost) function is represented in (1)^24,30^.

1 $$\:\mathbf{C}\mathbf{o}\mathbf{s}\mathbf{t}\left({\mathbf{I}}_{\varvec{j}}\right)=\sum\:_{\varvec{t}=1}^{\varvec{T}}\left\{\sum\:_{\varvec{k}=1}^{{\varvec{N}}_{\varvec{g}}}\left[{\varvec{Q}}_{\varvec{k}}\left(\varvec{t}\right){\varvec{E}}_{\varvec{g}\varvec{k}}\left(\varvec{t}\right){\varvec{P}}_{\varvec{g}\varvec{k}}\right(\varvec{t}\left)\right]+\left[{\varvec{Q}}_{\varvec{h}}\left(\varvec{t}\right){\varvec{E}}_{\varvec{B}\varvec{a}\varvec{t}}\left(\varvec{t}\right){\varvec{P}}_{\varvec{B}\varvec{a}\varvec{t}}\left(\varvec{t}\right)\right]\right\}$$

Where *Cost(I*_*j*_*)* is the cost value of the *j*^*th*^ individual; *j={1*,*2*,*…*,*n}*, *T* represents the total time in hours, and *N*_*g*_ is the power generation unit. The status of *N*_*g*_ and battery storage at time *t* are *Q*_*k*_*(t)* and *Q*_*h*_*(t)*, respectively, which may be in ON or OFF mode. *E*_*gk*_*(t)* is the amount of energy output by the generation units at time *t*; *E*_*gk*_*(t)=[E*_*PV*_*(t)*,* E*_*WIND*_*(t)*,* E*_*NGG*_*(t)]*. *E*_*Bat*_*(t)* is the amount of energy output by the battery storage devices at time *t. P*_*gk*_*(t)* is the price of energy provided for each generated unit at time *t*, while *P*_*Bat*_*(t)* is the price of energy provided for the battery storage devices at time *t*. *E*_*PV*_*(t)=[E*_*PV*_*(1)*,*E*_*PV*_*(2)*,*…*,*E*_*PV*_*(T)]*,*E*_*WIND*_*(t)=[E*_*WIND*_*(1)*,*E*_*WIND*_*(2)*,*…*,*E*_*WIND*_*(T)]*,*E*_*NGG*_*(t)=[E*_*NGG*_*(1)*,*E*_*NGG*_*(2)*,*…*,*E*_*NGG*_*(T)]*, and *E*_*Bat*_*(t)=[E*_*Bat*_*(1)*,*E*_*Bat*_*(2)*,*…*,*E*_*Bat*_*(T)]*. After applying the cost function to all individuals in the population, the best solution will be determined. Then, the switching mechanism (A) will be updated to switch between exploration and exploitation using (2).

To achieve this balance, ISCHO simulates the properties of Sinh and Cosh functions, allowing for effective search space exploration and convergence to the optimal solutions. A switching mechanism to move between the exploration and exploitation phases based on these nonlinear functions can be provided by Eq. (2). Thanks to this effectively approach, the algorithm can escape local minima and effectively explore the global search space.

2 $$\:\mathbf{A}=(\mathbf{q}-\mathbf{p}\times\:{\left(\frac{\varvec{t}}{\varvec{M}\varvec{a}{\varvec{x}}_{\varvec{t}}}\right)}^{\left(\frac{\mathbf{cosh}\frac{\varvec{t}}{\varvec{M}\varvec{a}{\varvec{x}}_{\varvec{t}}}}{\mathbf{sinh}\frac{\varvec{t}}{\varvec{M}\varvec{a}{\varvec{x}}_{\varvec{t}}}}\right)})\times\:{\varvec{r}}_{1}$$

Where *t* is the current iteration and *Max*_*t*_ is the maximum iterations number. The value of *r*_*1*_ was adjusted using a nonlinearly decreasing mode, and its change in value was calculated using a *cosh* function between 0 and π using (3).

3 $$\:{\varvec{r}}_{1}=\frac{{\varvec{\varnothing\:}}_{\varvec{m}\varvec{a}\varvec{x}}-{\varvec{\varnothing\:}}_{\varvec{m}\varvec{i}\varvec{n}}}{2}\mathbf{cosh}\left(\frac{{\uppi\:}\:\text{t}}{\varvec{M}\varvec{a}{\varvec{x}}_{\varvec{t}}}\right)+\frac{{\varvec{\varnothing\:}}_{\varvec{m}\varvec{a}\varvec{x}}+{\varvec{\varnothing\:}}_{\varvec{m}\varvec{i}\varvec{n}}}{2}$$

Where $\:{\varvec{\varnothing\:}}_{\varvec{m}\varvec{a}\varvec{x}}$ is the maximum value of inertia weight that equals 0.8 while $\:{\varvec{\varnothing\:}}_{\varvec{m}\varvec{i}\varvec{n}}$ is the minimum value of it that equals 0.75678. *t* is the current iteration, and *Max*_*t*_ is the maximum iterations number. To control the exploration and exploitation through iteration, *q* and *p* are the balance coefficients, which are equal to 10 and 9, respectively. In the case if *A > 1*, exploration will be performed, while exploitation will be performed in the case if *A < 1*. The current Boundary Search (BS) should be tested to determine if the search space will be updated or the weight coefficients (W_1_, W_2_, W_3_) based on exploration and exploitation will be updated. If *t* is equal to the *k*^*th*^ BS (BS_k_), the upper bound (ub_k_) and lower bound (lb_k_) of the potential search space will be updated using (4) and (5).

4 $$\:{\varvec{u}\varvec{b}}_{\varvec{k}}={\varvec{I}}_{\varvec{b}\varvec{e}\varvec{s}\varvec{t}}^{\left(\varvec{j}\right)}+(1-\frac{\varvec{t}}{{\varvec{M}\varvec{a}\varvec{x}}_{\varvec{t}}})\times\:|{\varvec{I}}_{\varvec{b}\varvec{e}\varvec{s}\varvec{t}}^{\left(\varvec{j}\right)}-{\varvec{I}}_{\varvec{s}\varvec{e}\varvec{c}\varvec{o}\varvec{n}\varvec{d}}^{\left(\varvec{j}\right)}|$$

5 $$\:{\varvec{l}\varvec{b}}_{\varvec{k}}={\varvec{I}}_{\varvec{b}\varvec{e}\varvec{s}\varvec{t}}^{\left(\varvec{j}\right)}-(1-\frac{\varvec{t}}{{\varvec{M}\varvec{a}\varvec{x}}_{\varvec{t}}})\times\:|{\varvec{I}}_{\varvec{b}\varvec{e}\varvec{s}\varvec{t}}^{\left(\varvec{j}\right)}-{\varvec{I}}_{\varvec{s}\varvec{e}\varvec{c}\varvec{o}\varvec{n}\varvec{d}}^{\left(\varvec{j}\right)}|$$

Where $\:{\varvec{I}}_{\varvec{b}\varvec{e}\varvec{s}\varvec{t}}^{\left(\varvec{j}\right)}$ and $\:{\varvec{I}}_{\varvec{s}\varvec{e}\varvec{c}\varvec{o}\varvec{n}\varvec{d}}^{\left(\varvec{j}\right)}$ refer to the *j*^*th*^ position of the best and second-best solutions. On the other hand, if *t* is not equal to *BS*_*k*_, the A value will be tested to switch between exploration and exploitation cases. If A < 1 (exploitation phase) and t < = T (total time), the weight coefficient (W_3_) will be updated using (6) and also the position of individuals in the population will be updated using (7). If A < 1 and t > T, the weight coefficient (W_2_) will be updated using (8) the position will be updated using (9).

6 $${W_{\text{3}}}={r_{\text{2}}} \times {a_{\text{1}}} \times \left( {{\text{cosh}}{r_{\text{3}}}+u \times {\text{sinh}}{r_{\text{3}}}} \right)$$

7 $$\:{\mathbf{I}}_{(\varvec{i},\varvec{j})}^{\varvec{t}+1}=\left\{\begin{array}{c}{\varvec{\varnothing\:}\varvec{I}}_{\varvec{b}\varvec{e}\varvec{s}\varvec{t}}^{\left(\varvec{j}\right)}+{\varvec{r}}_{4}\times\:{\varvec{W}}_{3}\times\:{\mathbf{I}}_{\left(\varvec{i},\varvec{j}\right)}^{\varvec{t}},\:\:\:\:\:\:\:\:\:\:{\varvec{r}}_{5}>0.5\\\:{\varvec{\varnothing\:}\varvec{I}}_{\varvec{b}\varvec{e}\varvec{s}\varvec{t}}^{\left(\varvec{j}\right)}-{\varvec{r}}_{4}\times\:{\varvec{W}}_{3}\times\:{\mathbf{I}}_{\left(\varvec{i},\varvec{j}\right)}^{\varvec{t}},\:\:\:\:\:\:\:\:\:\:{\varvec{r}}_{5}<0.5\end{array}\right.$$

8 $${W_{\text{2}}}={r_{\text{6}}} \times {a_{\text{2}}}$$

9 $$\:{\mathbf{I}}_{(\varvec{i},\varvec{j})}^{\varvec{t}+1}=\varvec{\varnothing\:}{\mathbf{I}}_{(\varvec{i},\varvec{j})}^{\varvec{t}}+{\varvec{r}}_{7}\times\:\frac{\text{s}\text{i}\text{n}\text{h}{r}_{8}}{\text{C}\text{o}\text{s}\text{h}{r}_{8}}|{{\varvec{W}}_{2}\times\:\varvec{I}}_{\varvec{b}\varvec{e}\varvec{s}\varvec{t}}^{\left(\varvec{j}\right)}-{\varvec{I}}_{(\varvec{i},\varvec{j})}^{\varvec{t}}|$$

Where *r*_*2*_, *r*_*3*_, *r*_*4*_, *r*_*5*_, *r*_*6*_, *r*_*7*_, and *r*_*8*_ are random values in [0,1] and *u* is equal to 0.388, such as in the first exploration phase. a_1_ and a_2_ are calculated using (10) and (11).

10 $$\:{\mathbf{a}}_{1}=3\times\:(-1.3\times\:\frac{\varvec{t}}{{\varvec{M}\varvec{a}\varvec{x}}_{\varvec{t}}}+\mathbf{m})$$

11 $$\:{\mathbf{a}}_{2}=2\times\:(-\frac{\varvec{t}}{{\varvec{M}\varvec{a}\varvec{x}}_{\varvec{t}}}+\mathbf{s})$$

Where *m* is equal to 0.45, that represents the sensitive coefficient to control the exploration accuracy. *s* is equal to 0.5, which represents the sensitive coefficient to control the exploration accuracy in the second phase. If A > 1 (exploration phase) and t < = T, the weight coefficient (W_1_) will be updated using (12), and also the position of individuals in the population will be updated using (13). If A > 1 and t > T, the weight coefficient (W_2_) will be updated using (8) and the position will be updated using (14).

12 $${W_{\text{1}}}={r_{\text{9}}} \times {a_{\text{1}}} \times \left( {{\text{cosh}}{r_{{\text{1}}0}}+u \times {\text{sinh}}{r_{{\text{1}}0}} - {\text{1}}} \right)$$

13 $$\:{\mathbf{I}}_{(\varvec{i},\varvec{j})}^{\varvec{t}+1}=\left\{\begin{array}{c}{\varvec{\varnothing\:}\varvec{I}}_{\varvec{b}\varvec{e}\varvec{s}\varvec{t}}^{\left(\varvec{j}\right)}+{\varvec{r}}_{11}\times\:{\varvec{W}}_{1}\times\:{\mathbf{I}}_{\left(\varvec{i},\varvec{j}\right)}^{\varvec{t}},\:\:\:\:\:\:\:\:\:\:{\varvec{r}}_{12}>0.5\\\:\varnothing\:{\varvec{I}}_{\varvec{b}\varvec{e}\varvec{s}\varvec{t}}^{\left(\varvec{j}\right)}-{\varvec{r}}_{11}\times\:{\varvec{W}}_{1}\times\:{\mathbf{I}}_{\left(\varvec{i},\varvec{j}\right)}^{\varvec{t}},\:\:\:\:\:\:\:\:\:\:{\varvec{r}}_{12}<0.5\end{array}\right.$$

14 $$\:{\mathbf{I}}_{(\varvec{i},\varvec{j})}^{\varvec{t}+1}=\left\{\begin{array}{c}{\varvec{\varnothing\:}\varvec{I}}_{(\varvec{i},\varvec{j})}^{\varvec{t}}+|\epsilon\:\times\:{\varvec{W}}_{2}\times\:{\varvec{I}}_{\varvec{b}\varvec{e}\varvec{s}\varvec{t}}^{\left(\varvec{j}\right)}-{\varvec{I}}_{(\varvec{i},\varvec{j})}^{\varvec{t}}|,\:\:\:\:\:\:\:\:\:\:{\varvec{r}}_{13}>0.5\\\:\varnothing\:{\varvec{I}}_{(\varvec{i},\varvec{j})}^{\varvec{t}}-|\epsilon\:\times\:{\varvec{W}}_{2}\times\:{\varvec{I}}_{\varvec{b}\varvec{e}\varvec{s}\varvec{t}}^{\left(\varvec{j}\right)}-{\varvec{I}}_{(\varvec{i},\varvec{j})}^{\varvec{t}}|,\:\:\:\:\:\:\:\:\:\:{\varvec{r}}_{13}<0.5\end{array}\right.$$

Where *r*_*9*_, *r*_*10*_, *r*_*11*_, *r*_*12*_, and *r*_*13*_ are random values in [0,1] and ε is a tiny positive number that equals 0.003. These steps will be continued until *t* is equal to *Max*_*t*_. At the end, the best solution is one that gives the minimum cost value. $\:\varvec{\varnothing\:}$ is the inertia weight that represents an adaptive variable that will decrease when the number of iterations is increased. $\:\varvec{\varnothing\:}$ can be calculated using (15).

15 $$\:{\varvec{\varnothing\:}}_{\varvec{t}}={\varvec{\varnothing\:}}_{\varvec{m}\varvec{a}\varvec{x}}-({\varvec{\varnothing\:}}_{\varvec{m}\varvec{a}\varvec{x}}-{\varvec{\varnothing\:}}_{\varvec{m}\varvec{i}\varvec{n}})\times\:\frac{\varvec{t}}{{\varvec{M}\varvec{a}\varvec{x}}_{\varvec{t}}\:}$$

At the end, energy sources can be scheduled depending on the optimized results from ISCHO, and the customer can determine the best source based on the current prices.

## Simulation and results

In this section, a new optimizer called **ISCHO** used to optimize energy management systems will be tested and compared to other recent optimizers to prove its efficiency against them. These optimizers are SCHO^14^, ISC^15^, Chimp^16^, IGWO^17^, and COA^18^. The proposed ISCHO will be executed against these five algorithms using five benchmark functions for ensuring the superiority of ISCHO against standard SCHO and other recent algorithms (SCHO, ISC, Chimp, IGWO, and COA). After that, the proposed ISCHO will be executed to optimize the energy management system as a case study through three scenarios depending on three different maximum iteration numbers (Max_t_ =200, 500, and 1000). In each scenario, the implementation will be performed according to two different population sizes (*n* = 500 and 1000). The initial population depends on KU-HMG dataset^31^.

The initial values for all optimizers’ candidate solutions are derived from the “Payra Original load.csv” file within the KU-HMG dataset. Each solution represents the levels of four energy sources: battery state of charge (%), photovoltaic power output (kW), wind turbine power output (kW), and generator power output (kW), all of which are specified in the dataset. Utilizing real-world data for the initial dataset, instead of generating it randomly, aims to decrease the optimizers’ runtime and yield more precise solutions.

There are many performance metrics called Fitness value (F), Execution Time (ET), mean (Mean), and Standard Deviation (Std.)^15,18^. All algorithms have a number of common parameters, which are listed in Table 1. All algorithms depend on the same number of iterations (Max_t_) and also the same number of search agents or population size (n). Additionally, *r* refers to a random value between [0,1]. Furthermore, a statistical analysis will be conducted to compare the ISCHO algorithm with other algorithms, employing the following metrics and tests: Mean, Std., Confidence Intervals (CI), t-test (with a significance level of 0.05), and Wilcoxon test (with a significance level of 0.05)^32,33^. Table 2 displays the parameter values that were used for each optimization algorithm during execution. The tests were conducted on the same platform, according to the simulation platform, where all algorithms were evaluated. The simulations were conducted using MATLAB R2018a (Version 9.4), MathWorks, Natick, MA, USA (https://www.mathworks.com/products/matlab.html). This software was loaded on a laptop running Intel (R) Core (TM) i5-10210U and Windows 10 (64 bit).

**Table 1:** The common parameters and their values in implementation.

Parameter	Description	Assigned value
Max_t_	Number of iterations	200, 500, 1000
n	Number of search agents	500, 1000
r	Random value	[0,1]

According to Table 2, the values of the parameters ( Ct, u ,m,$\:\:\varvec{\epsilon\:}$, s, q, p, $\:\varvec{\alpha\:}$, and$\:\:\varvec{\beta\:}$) for the ISCHO were used in the same original method, which is SCHO, for the accuracy of comparing ISCHO with SCHO and other methods, while the values of the $\:{\varvec{\varnothing\:}}_{\varvec{m}\varvec{a}\varvec{x}}$ and $\:{\varvec{\varnothing\:}}_{\varvec{m}\varvec{i}\varvec{n}}$ were determined through practical experiments.

**Table 2:** The used values of parameters of optimizers during execution.

Optimizer	Parameter	Value
ISCHO	Ct, u, m	3.6, 0.388, 0.45
	$\:\varvec{\epsilon\:}$, s	0.003, 0.5
	q, p	10, 9
	$\:\varvec{\alpha\:},\:\:\varvec{\beta\:}$	1.6, 1.55
	$\:{\varvec{\varnothing\:}}_{\varvec{m}\varvec{a}\varvec{x}}$, $\:{\varvec{\varnothing\:}}_{\varvec{m}\varvec{i}\varvec{n}}$	0.8, 0.75678
SCHO^14^	Ct, u, m	3.6, 0.388, 0.45
	$\:\varvec{\epsilon\:}$, n	0.003, 0.5
	p, q	10, 9
	$\:\varvec{\alpha\:},\:\:\varvec{\beta\:}$	1.6, 1.55
ISC^15^	$\:\varvec{\alpha\:}$(constant)	2
Chimp^16^	m	Chaotic
IGWO^17^	The encircling coefficient (a)	From 2 to 0
COA^18^	I(random value)	[1, 2]

### The description of KU-HMG dataset

A hybrid AC-DC micro-grid has been constructed and simulated with the goal of providing an affordable electrical supply for the Payra region of Bangladesh (22.1493° N, 90.1352° E)^31^. The popular simulation program “Homer Pro” was used for the design. Within the micro-grid, 30 DC loads from electric vehicles and 100 AC loads from households made up the consumers, while solar photovoltaic panels, wind turbines, and natural gas generators handled the sources. Wind speed, hourly yearly load demand, and solar global horizontal irradiance were the matching input parameters. The load demand has been roughly replicated by raising and lowering it by 10%, 5%, and 2.5% more and less, respectively, in order to make use of the generated electricity. In conclusion, seven datasets emerge, comprising the initial and six different ones. Our work depends on the “Payra_Original_load.csv” file that includes raw output data that was gathered from the micro-grid simulation and matches Payra’s initial load requirement. This dataset includes 8761 data instances in the dataset overall, with 14 data samples per set. The 14 data samples are called time, Photovoltaic panel Power Output (kW), Wind Turbine Power Output (kW), Generator Power Output (kW), Generator Fuel (m), Total Electrical Load Served (kW), Renewable Penetration (%),Excess Electrical Production (kW), Total Renewable Power Output (kW), Inverter Power Output (kW), Rectifier Power Output (kW), Battery Charge Power (kW), Battery Discharge Power (kW), and Battery State of Charge (%). In this work, the execution of algorithms depends on using 4 data samples called Photovoltaic panel Power Output, Wind Turbine Power Output, Generator Power Output, and Battery Discharge Power as shown in Table 3.

**Table 3:** A snapshot of the used dataset.

Time	Photovoltaic panel power output (kW)	Wind turbine power output (kW)	Generator power output (kW)	Battery state of charge (%)
1/1/2007 0:00	0	0	0	98.20293
1/1/2007 1:00	0	0	0	96.46697
1/1/2007 2:00	0	0	0	93.91097
1/1/2007 3:00	0	0	0	90.7637
1/1/2007 4:00	0	0	0	87.60335
1/1/2007 5:00	0	0	0	85.91619
1/1/2007 6:00	0	0	0	85.6179
1/1/2007 7:00	7.872308	0	0	85.80567
1/1/2007 8:00	14.0846	5.639925	0	86.61595
1/1/2007 9:00	25.70485	6.800035	0	88.06362
1/1/2007 10:00	6.040092	12.61806	0	88.75788
1/1/2007 11:00	4.564925	20.94857	0	89.70627
1/1/2007 12:00	23.20347	11.27311	0	91.22481
1/1/2007 13:00	19.52974	5.576788	0	92.32033

### The used benchmark functions

In this subsection, five objective (benchmark) functions used to evaluate the proposed ISCHO against other algorithms will be described. These five benchmark functions, labeled from F1 to F5, will be described with their minimum fitness values (F_min_), normal range, and dimension in Table 4^32,33^. In fact, a variety of metaheuristic optimization algorithms can be used in wide-range domains with small dimensions. On the other hand, many of these algorithms can be applied for small-range domains with big dimensions to solve many critical problems like feature selection. In fact, ISCHO can be used with these two types of domains, including discrete and continuous tasks.

**Table 4:** The used functions.

	Function	Range	Dimension	$\:{F}_{min}$
F1	$\:{F}_{1}\left(x\right)=\sum\:_{i=1}^{m}\left|{x}_{i}\right|+\prod\:_{i=1}^{m}\left|{x}_{i}\right|$	[− 10, 10]	5	0
F2	$\:{F}_{2}\left(x\right)=\sum\:_{i=1}^{m}{\left(\left[{x}_{i}+.5\right]\right)}^{2}$	[− 100, 100]	5	0
F3	$$\begin{aligned} \:F_{3} \left( x \right) & = [(1 + (x_{1} + x_{2} )^{2} )(19 - 14x_{1} + 3x_{1}^{2} - 14x_{2} + 6x_{1} x_{2} \\ & \quad + 3x_{2}^{2} )]*[((30 + (2x_{1} - 3x_{2} )^{2} )(18 - 32x_{1} + 12x_{1}^{2} + 48x_{2} \\ & \quad - 36x_{1} x_{2} + 27x_{2}^{2} )] \\ \end{aligned}$$	[− 5, 5]	2	3
F4	$\:{\text{F}}_{4}\left(\text{x}\right)={max}_{i}\{\left|x\right|,1\le\:i\le\:m$	[− 50, 50]	2	0
F5	$\:\text{F}5\left(\text{x}\right)=1-\text{cos}(2\pi\:\sqrt{{\sum\:}_{i=1}^{m}{x}_{i}^{2}})+0.1\sqrt{{\sum\:}_{i=1}^{m}{x}_{i}^{2}}$	[− 50, 50]	2	0

The five objective functions (F1–F5) in Table 4 were chosen as standard functions to test the performance of the proposed ISCHO against other algorithms. In fact, these functions are used in optimization research to evaluate the ability of algorithm to handle various types of optimization problems. ISCHO is designed to be applicable to both discrete and continuous problems and can be used with small-dimension, wide-range domains as well as large-dimension, small-range domains. Table 4 includes the details on their minimum fitness values (F_min_), normal range, and dimension. For example, F1 has a dimension of 5 and a range of [-10, 10] with a Fmin of 0. With a dimension of 5 and a F_min_ of 0, the range of F2 is [-100, 100]. These objective functions can simulate different difficulties that may arise in optimizing energy usage and minimizing operating costs in Nano-grids. Thus, these functions are relevant for the energy management problem because they offer a generalized evaluation of the performance of algorithm across a range of problem complexities. Using these objective functions, ISCHO performed better than other algorithms, demonstrating its strong performance.

### Testing ISCHO in a comparison to other algorithms using benchmark functions

In this subsection, the proposed ISCHO will be tested and compared to the other five algorithms presented in Table 2 using the five functions (F1 to F5) presented in Table 4. Table 5 tests and compares the ISCHO, SCHO, ISC, Chimp, IGWO, and COA.

**Table 5:** Results of algorithms based on the five functions (F1 to F5).

Algorithm	Fitness value				
	F1	F2	F3	F4	F5
ISCHO	0	0.165	− 1.0087	3.70	0.0654
SCHO	0.0796	0.0487	− 1.0031	3.954	0.0096
ISC	0.0856	0.0151	− 1.0074	3.363	0.0219
Chimp	0.0994	0.0871	− 1.0059	3.256	0.00787
IGWO	0.1895	0.0456	− 1.0073	3.089	0.0646
COA	0.0565	0.0225	− 1.0053	3.536	0.08

Based on the results in Table 5, it is ensured that ISCHO has strong performance across the five benchmark functions (F1 to F5). Notably, for function F1, ISCHO achieves the global minimum value of 0, a result that none of the other algorithms (SCHO, ISC, Chimp, IGWO, and COA) are able to replicate. Although ISCHO cannot produce the absolute minimum values for the remaining functions (F2, F3, F4, and F5), its obtained fitness values are generally competitive with, and in some cases better than, those achieved by the other algorithms. For instance, for F2, ISCHO’s value of 0.165 is higher than the minimum of 0.0151 achieved by ISC, but it outperforms SCHO, Chimp, IGWO, and COA. Similarly, for F3, ISCHO’s value of -1.0087 is close to the best value of -1.0074 given by ISC. In general, according to the five functions (F1 to F5), ISCHO demonstrates a robust ability to determine near-optimal solutions, particularly highlighted by its unique success in reaching the global minimum for F1, suggesting a potential advantage in its search mechanism compared to the other evaluated algorithms.

### Case study: testing ISCHO for optimizing energy management systems

In the following subsections, three main scenarios for executing ISCHO compared to the other algorithms for optimizing energy management systems will be introduced using different iteration numbers (200, 500, and 1000) using population sizes (500 and 1000). Based on population sizes (500 and 1000), the proposed ISCHO has the ability to achieve optimal results, specifically a fitness value, mean, and standard deviation of 0, which significantly surpassed other tested algorithms. The larger population sizes contributed to ISCHO’s consistent outperformance according to many different metrics, providing optimal energy values. Although population sizes are large, the competitive execution time of ISCHO asserting its effectiveness for real-time energy management in Nano-grids. While some algorithms might exhibit slightly faster execution times, ISCHO outperformed other algorithms because it can give optimal results which makes it a practical solution for real-time applications.

#### Testing ISCHO algorithm against other algorithms using *Max*_*t*_ = 200

**Fig. 4:**
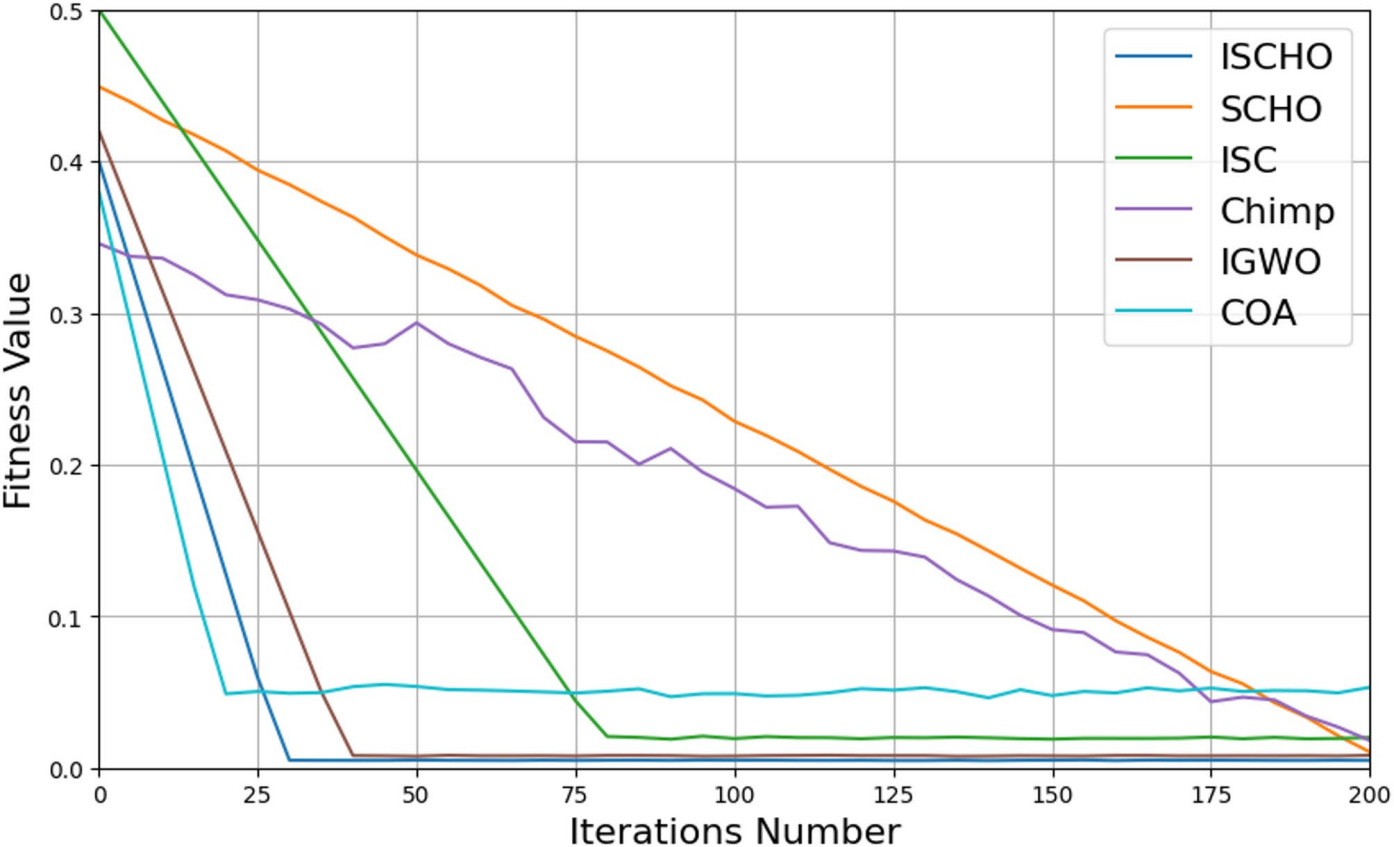
The fitness values of optimizers using Max_t_ = 200 and *n* = 500.

**Fig. 5:**
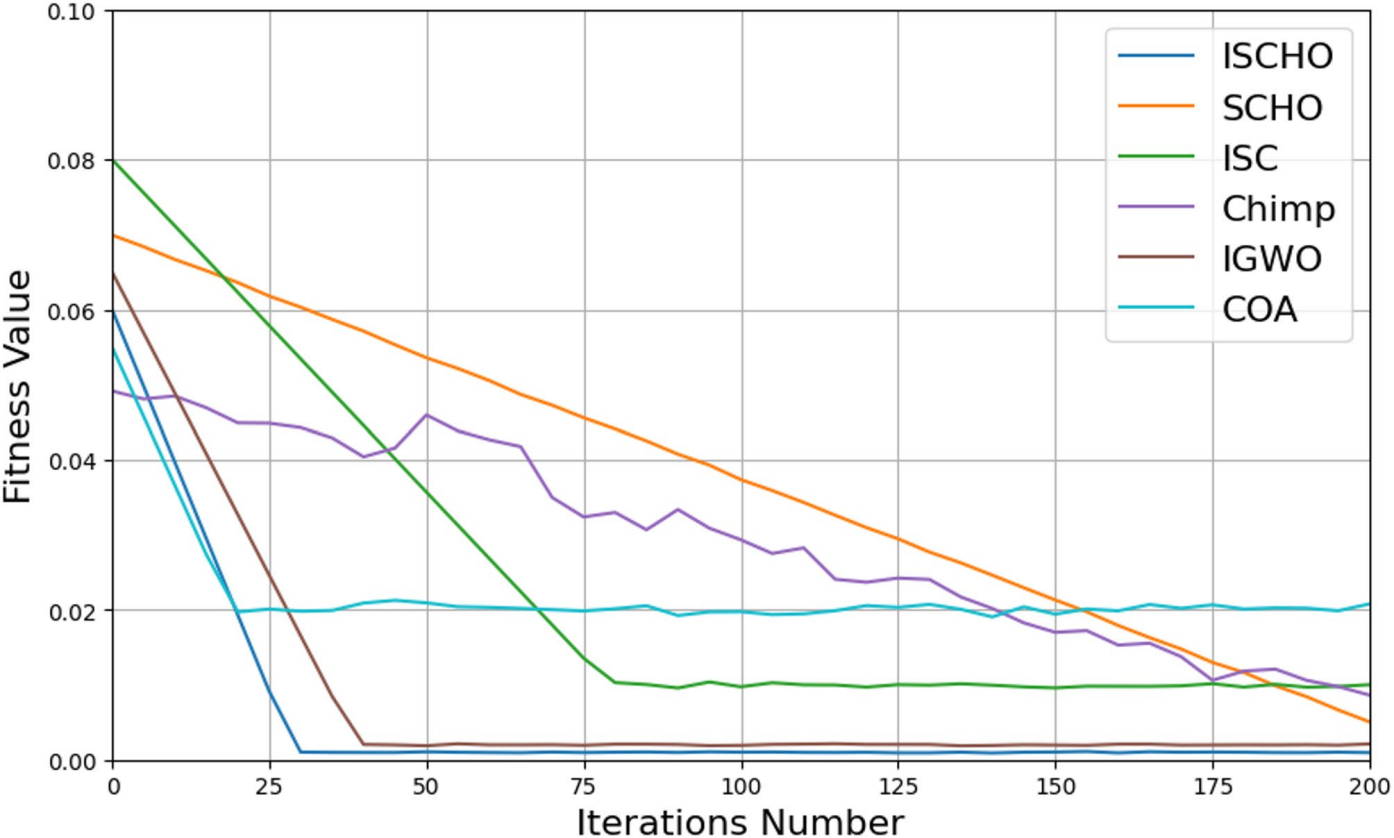
The fitness values of optimizers using Max_t_ = 200 and *n* = 1000.

**Fig. 6:**
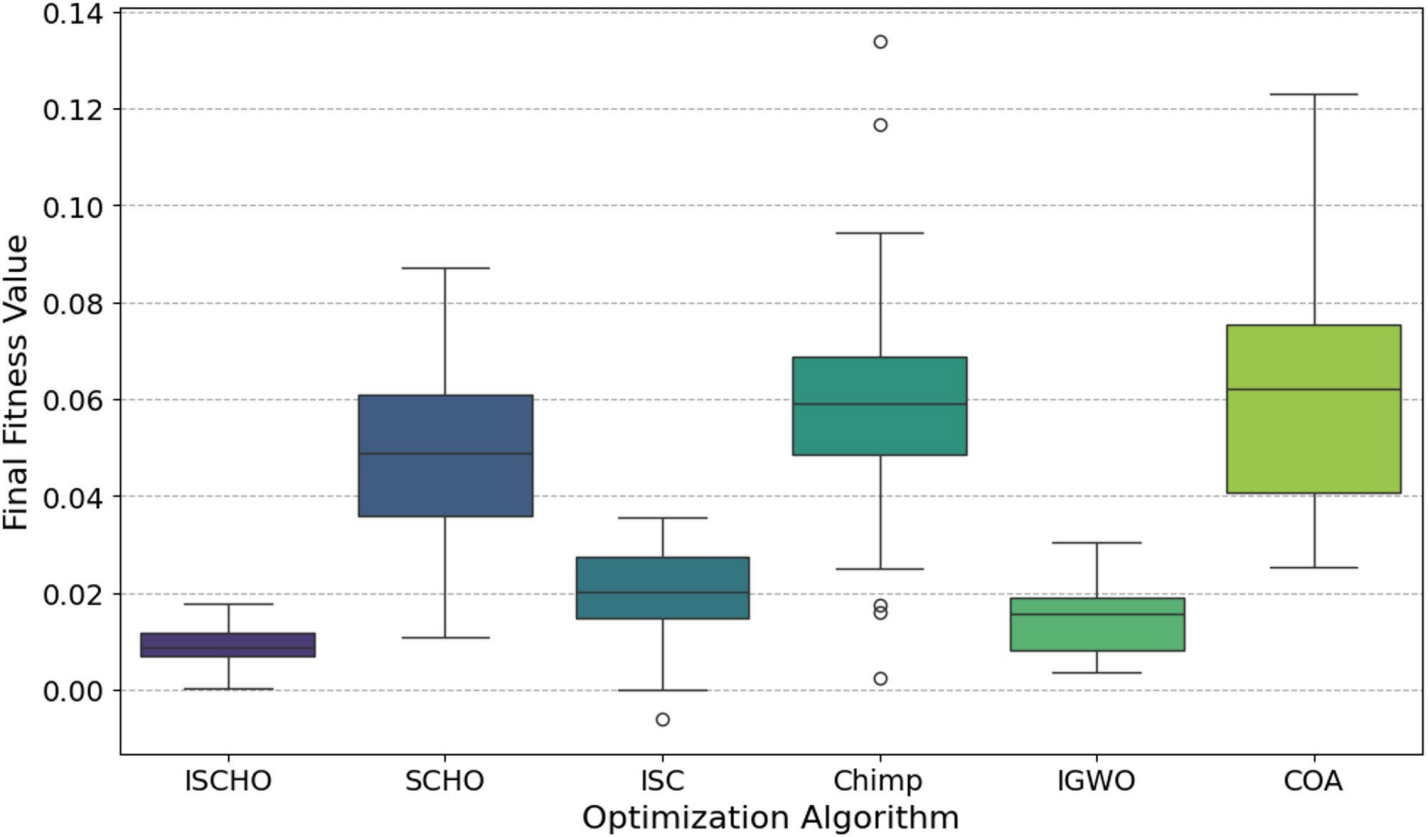
The Boxplot of the cost function across independent runs using Max_t_ = 200 and *n* = 500.

**Fig. 7:**
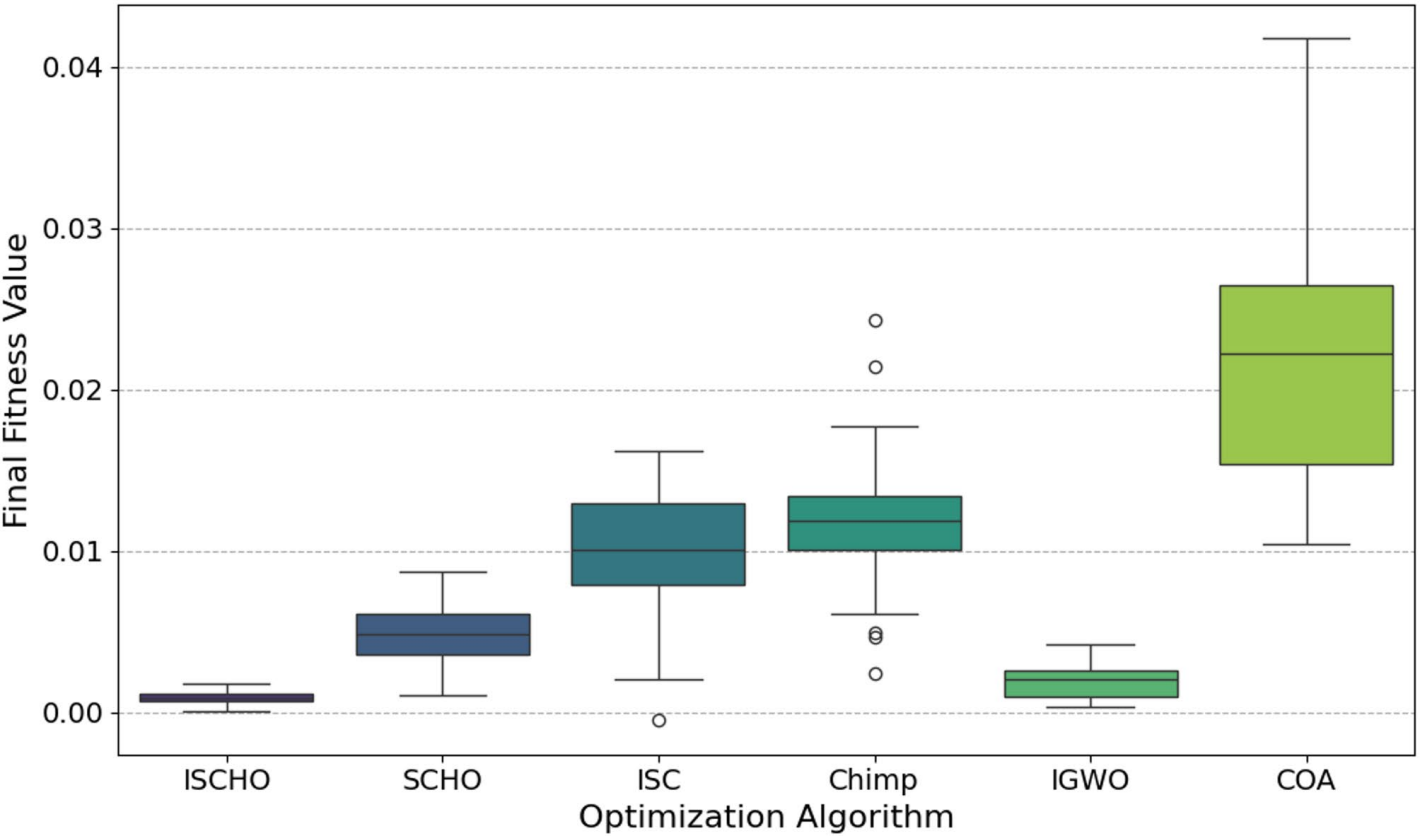
The Boxplot of the cost function across independent runs using Max_t_ = 200 and *n* = 500.

In this section, ISCHO will be tested and compared to other optimizers using n= (500 and 1000) at Max_t_ =200. Figures 4 and 5 present the fitness values of all optimizers according to each iteration number using *n* = 500 and 1000 respectively. Figures 6 and 7 represent the boxplot of the fitness (cost) function across independent runs (iterations) for the proposed ISCHO against other algorithms using Max_t_ =200 and n= (500 and 1000). Table 6 includes a comparison between optimizers at n= (500 and 1000) and Max_t_ =200 using F, ET (second unit), Mean, and Std. measurements.

**Table 6:** A comparison between optimizers at n= (500 and 1000) and Max_t_ = 200.

Algorithm	Metrics							
	*n* = 500				*n* = 1000			
	F*10^−6^	ET	Mean*10^−5^	Std.	F*10^−6^	ET	Mean*10^−5^	Std.
ISCHO	0	3.00862	0	0	0	3.3287	0	0
SCHO	4.1517	3.95806	3.1617	0	16.705	6.9513	2.0705	0
ISC	13.895	9.82959	3.3995	0	11.811	13.826	1.1625	0
Chimp	23.768	2.1627	2.8968	0	17.586	2.9765	10.2506	0
IGWO	0.0024137	20.6888	0.025247	0	0.0015933	30.3309	0.010933	0
COA	0.0003	3.638858	0.0005	0	0.00001	5.0235	0.0002	0

Depending on results provided in Figs. 4 and 6, and Table 6, it is noted that ISCHO outperformed SCHO, ISC, Chimp, IGWO, and COA at a population size equal to 500 as it provided the minimum fitness value, Mean, and Std. (F = 0, Mean = 0, Std.=0). Hence, the first best optimizer is ISCHO which can provide the optimal energy values. The second-best optimizer after ISCHO is COA, as it can provide the best F, Mean, Std. values after ISCHO, and the third-best optimizer is IGWO. On the other hand, the worst optimizer is Chimp, although it consumed the minimum execution time because it provided the maximum F and Mean (F = 23.768*10^− 6^, Mean = 2.8968*10^− 5^).

Figures 5 and 7, and Table 6 show that the performance of ISCHO is superior to other optimizers at a population size equal to 1000. Accordingly, the first best optimizer is ISCHO, the second best one is COA, and the third best one is IGWO. On the other hand, the worst optimizer that provided the maximum F and Mean values is Chimp. In fact, Std. value is zero for all optimizers at both population sizes (*n* = 500 and *n* = 1000). According to ET values at n equal to 500 and 1000, the maximum execution time is provided by IGWO while the minimum execution time is provided by Chimp. Although Chimp’s execution time is lower than ISCHO, the efficiency of ISCHO is better than Chimp because it provided the best F and Mean values.

For *n* = 500 (Fig. 6), ISCHO exhibits the lowest median fitness value and the smallest interquartile range, indicating high consistency and robustness in achieving optimal or near-optimal cost functions. Additionally, COA and IGWO give the minimum fitness values and good consistency. On the other hand, SCHO, ISC, and Chimp provide the maximum median fitness values and larger spreads, proposing less consistent performance and higher final cost functions. Notably, Chimp shows the maximum median fitness value, indicating it is the worst performer in this scenario.

Likewise, ISCHO maintains its strong performance with the lowest median fitness value and the least amount of spread for *n* = 1000 (Fig. 7), confirming its stability and reliability at a higher population size. Moreover, COA and IGWO continue to compete competitively. Conversely, the boxplots for SCHO, ISC, and Chimp once more show greater and less consistent fitness values than ISCHO, COA, and IGWO. Among all optimizers, Chimp continues to get the worst results.

At the end, based on the results from Figs. 4, 5, 6 and 7, and Table 6, ISCHO consistently outperformed other optimizers across both population sizes (*n* = 500 and *n* = 1000), providing the minimum F, Mean, and Std., and thus offering optimal energy values. COA and IGWO were the next best performers, while Chimp consistently ranked as the worst optimizer in terms of fitness and mean values.

#### Testing ISCHO algorithm against other algorithms using *Max*_*t*_ = 500

**Fig. 8:**
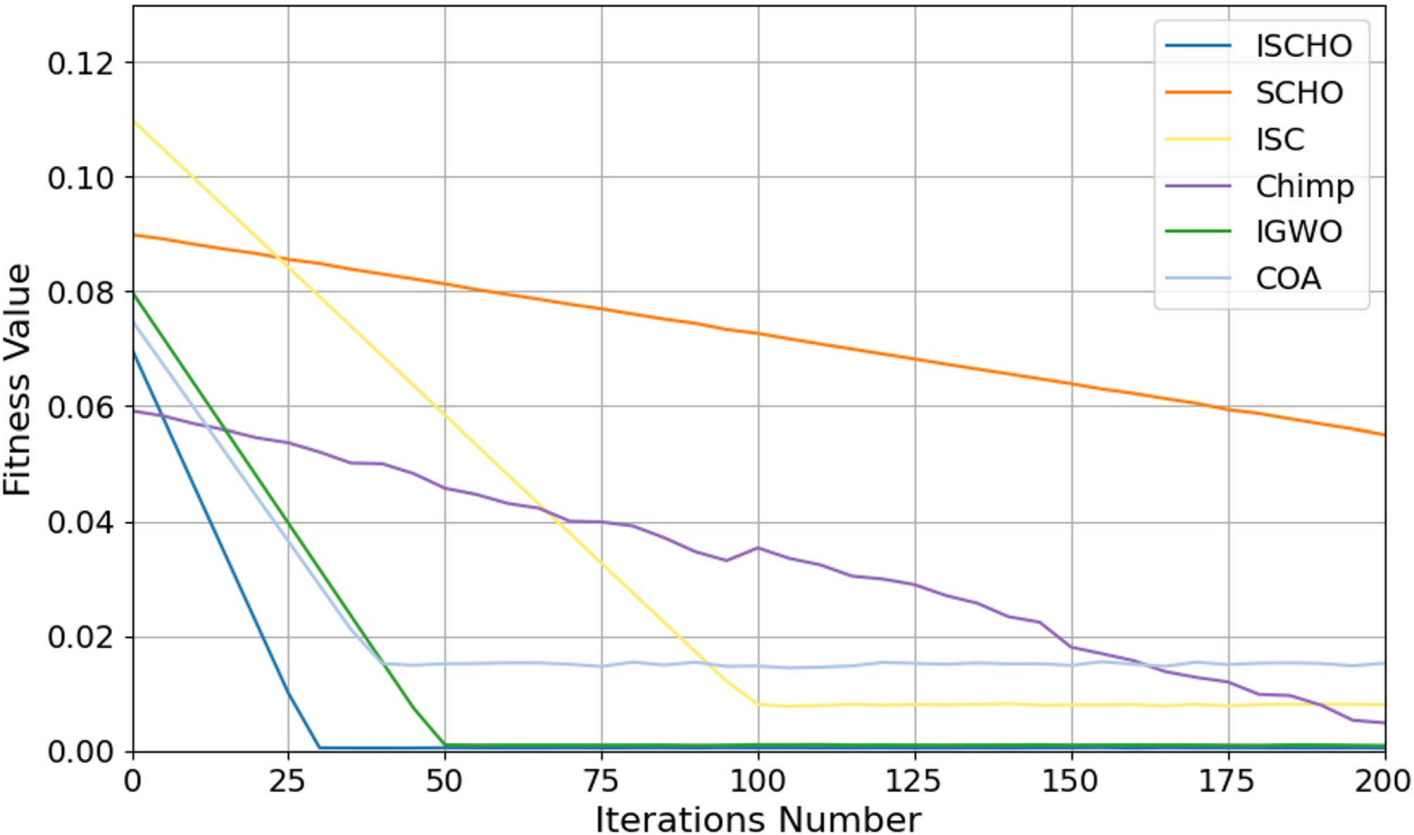
The fitness values of optimizers using Max_t_ = 500 and *n* = 500.

**Fig. 9:**
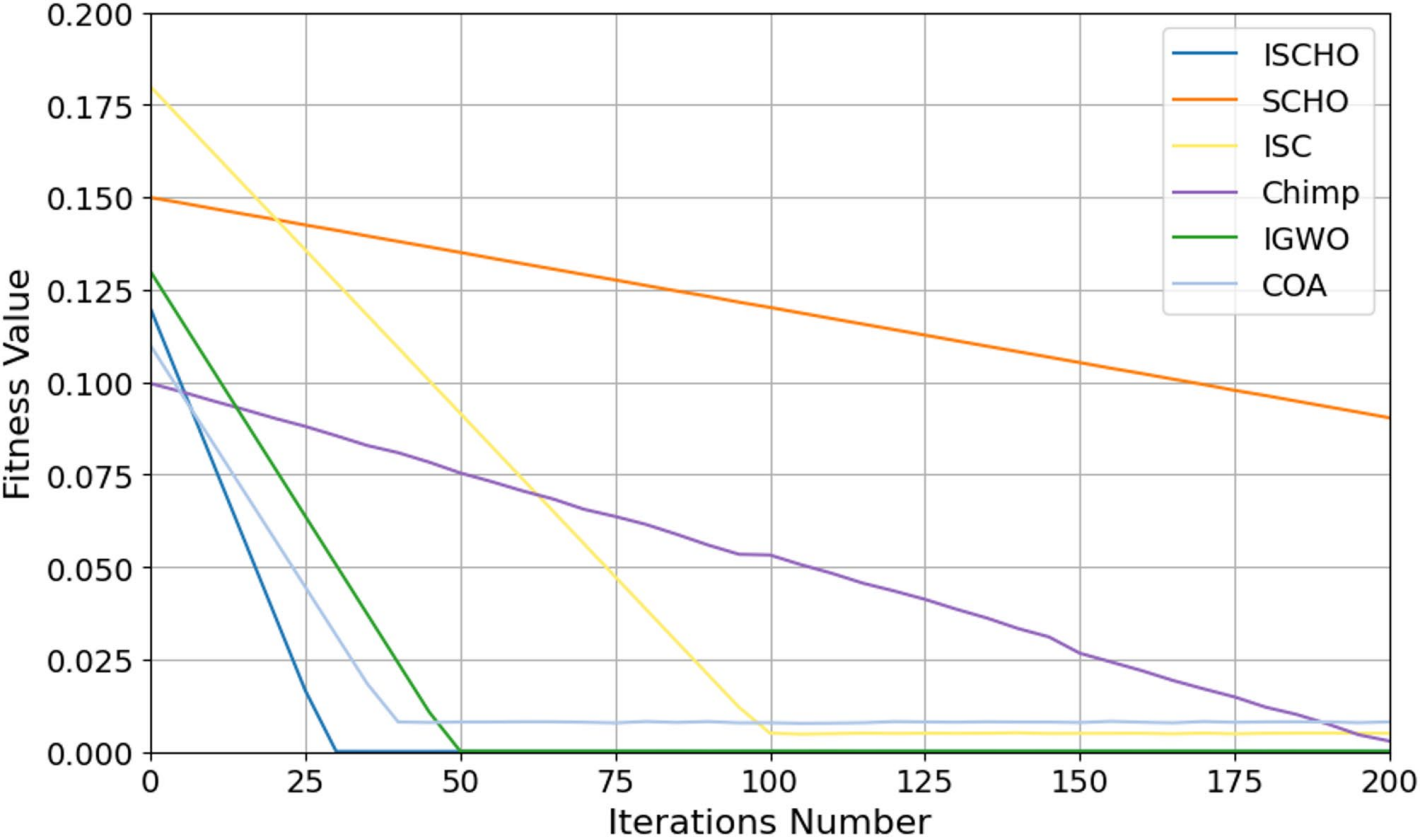
The fitness values of optimizers using Max_t_ = 500 and *n* = 1000.

**Fig. 10:**
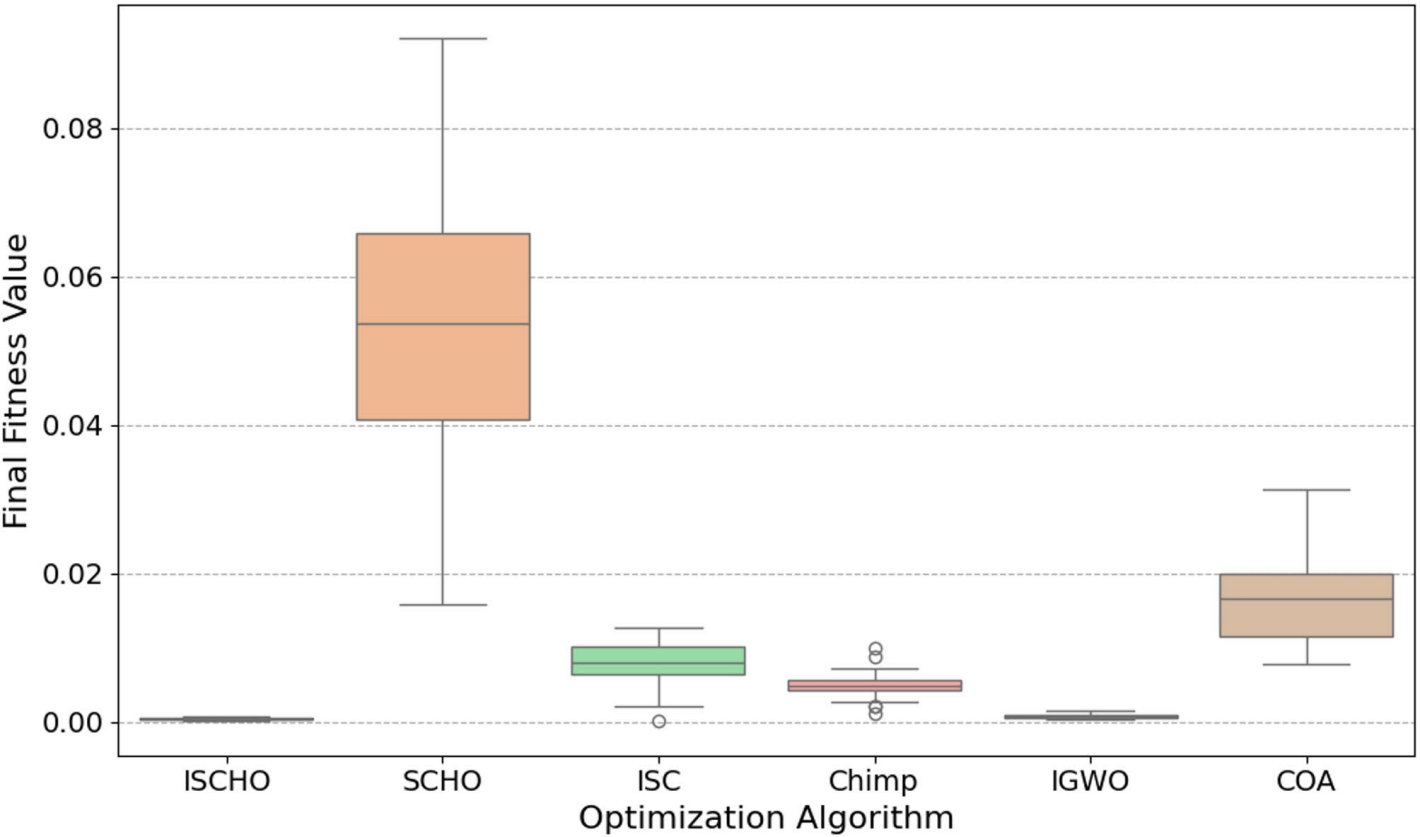
The Boxplot of the cost function across independent runs using Max_t_ = 500 and *n* = 500.

**Fig. 11:**
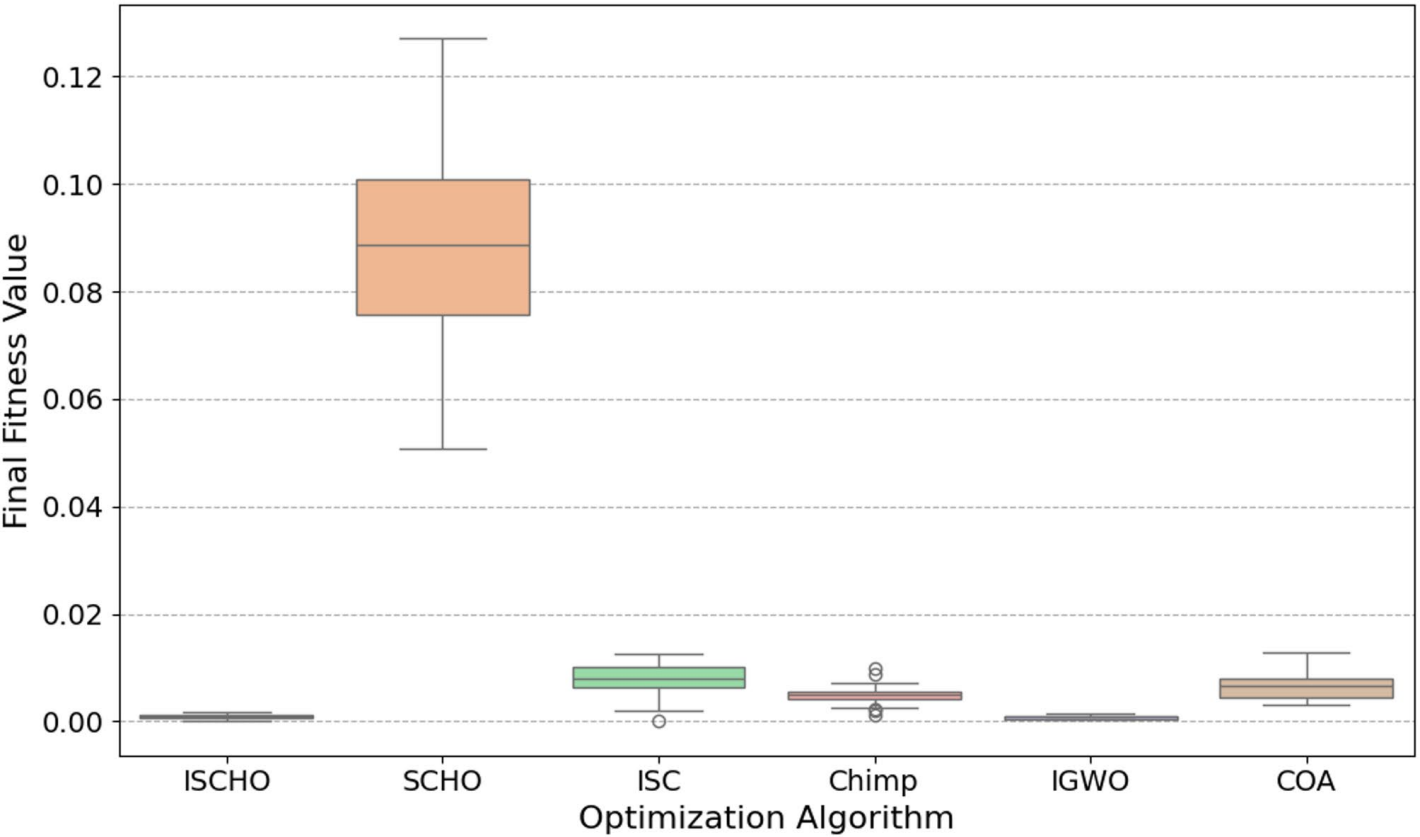
The Boxplot of the cost function across independent runs using Max_t_ = 500 and *n* = 1000.

In this section, ISCHO will be tested and compared to other optimizers using n= (500 and 1000) at Max_t_ =500. Figures 8 and 9 represent the fitness values of all optimizers according to each iteration number, using *n* = 500 and 1000, respectively. Figures 10 and 11 represent the boxplot of the fitness (cost) function across independent runs (iterations) for the proposed ISCHO against other algorithms using Max_t_ =500 and n= (500 and 1000). Table 7 includes a comparison between optimizers at n= (500 and 1000) and Max_t_ =500 using F, ET (second unit), Mean, and Std. measurements.

**Table 7:** A comparison between optimizers at n = (500 and 1000) and Max_t_ = 500.

Algorithm	Metrics							
	*n* = 500				*n* = 1000			
	F*10^−6^	ET	Mean*10^−6^	Std.	F*10^−6^	ET	Mean*10^−6^	Std.
ISCHO	0	4.40862	0	0	0	5.11015	0	0
SCHO	11.082	4.96606	11.082	0	3.629	6.58637	3.629	0
ISC	7.4924	11.92559	7.4924	0	0.73211	17.1157	0.732111	0
Chimp	9.6852	3.1727	9.6852	0	5.7985	4.80286	5.7985	0
IGWO	0.00017434	26.6498	0.00017434	0	0.0000782	90.37017	0.0000782	0
COA	0.00007	5.638858	0.00007	0	0.0000005	10.08509	0.00000005	0

It is noted that the F and Mean values for all optimizers presented in Table 7 (at Max_t_=500) are better than their values provided in Table 7 (at Max_t_=200) according to n equals 500 and 1000. On the other hand, the execution time is increased for all optimizers when Max_t_ is increased. The results of Figs. 8 and 10, and Table 7 illustrate that ISCHO outperformed SCHO, ISC, Chimp, IGWO, and COA at n equals 500 because it provided zero value for F, Mean, and Std. Thus, the first best optimizer is ISCHO, the second best optimizer after ISCHO is COA, and the third best optimizer is IGWO. On the other hand, the worst optimizer is SCHO because it provided the maximum F and Mean (F = 11.082*10^− 6^, Mean = 11.082*10^− 6^).

Figures 9 and 11, and Table 7 proved that ISCHO is superior to other optimizers at n equals 1000. Thus, the first best optimizer is ISCHO, the second best one is COA, and the third best one is IGWO. On the other hand, the worst optimizer that provided the maximum F and Mean values is ISC. In all cases, Std. value is zero for all optimizers at both population sizes (*n* = 500 and *n* = 1000). According to ET values at n equal to 500 and 1000, the maximum execution time is provided by IGWO while the minimum execution time is provided by Chimp. Although Chimp’s execution time is lower than ISCHO, the efficiency of ISCHO is better than Chimp because it provided the best F and Mean values.

Finally, the analysis of Figs. 8, 9, 10 and 11; Table 7 shows that ISCHO consistently demonstrates superior performance across both population sizes (*n* = 500 and *n* = 1000). It consistently provides zero or near-zero fitness values (F) and mean values (Mean), indicating optimal or near-optimal cost functions and high consistency. Also, the boxplots provide that ISCHO has extraordinary robustness and dependability with the lowest median fitness values and minimal interquartile ranges. With competitive convergence and strong constancy in their fitness values, COA and IGWO are the next best optimizers. The IGWO’s performance is strong in terms of fitness values, although it shows slightly longer execution times compared to ISCHO and COA. On the other hand, the boxplots of SCHO, ISC, and Chimp consistently present bigger spreads and higher median fitness values, referring to less consistent performance and higher final cost functions. It is noted that Chimp is the worst optimizer in terms of fitness and mean values, even though it has the shortest execution time. ISC performs rather poorly as well, particularly when *n* = 1000. Interestingly, the standard deviation (Std.) for all optimizers is consistently zero for both population sizes.

#### Testing ISCHO algorithm against other algorithms using *Max*_*t*_ = 1000

**Fig. 12:**
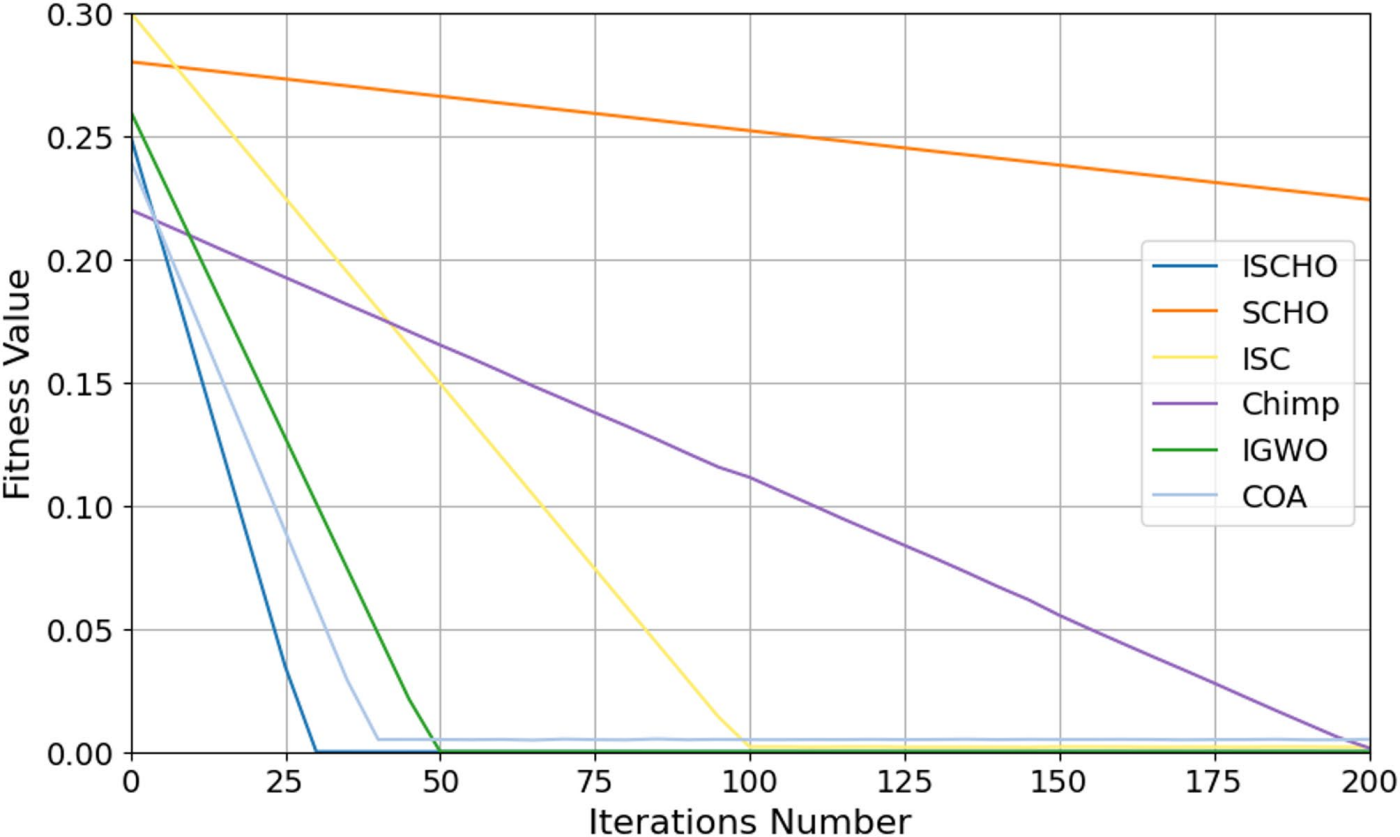
The fitness values of optimizers using Max_t_ = 1000 and *n* = 500.

**Fig. 13:**
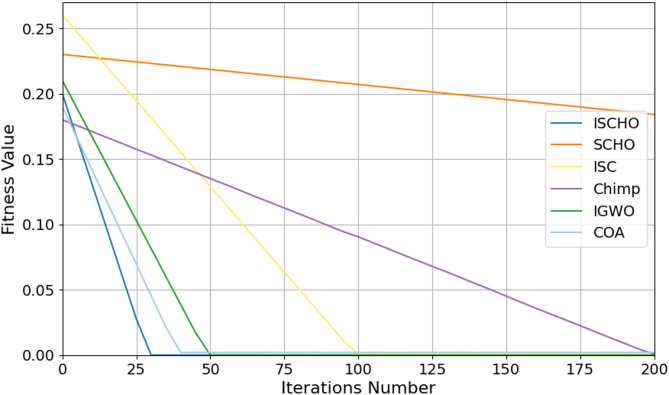
The fitness values of optimizers using Max_t_ = 1000 and *n* = 1000.

**Fig. 14:**
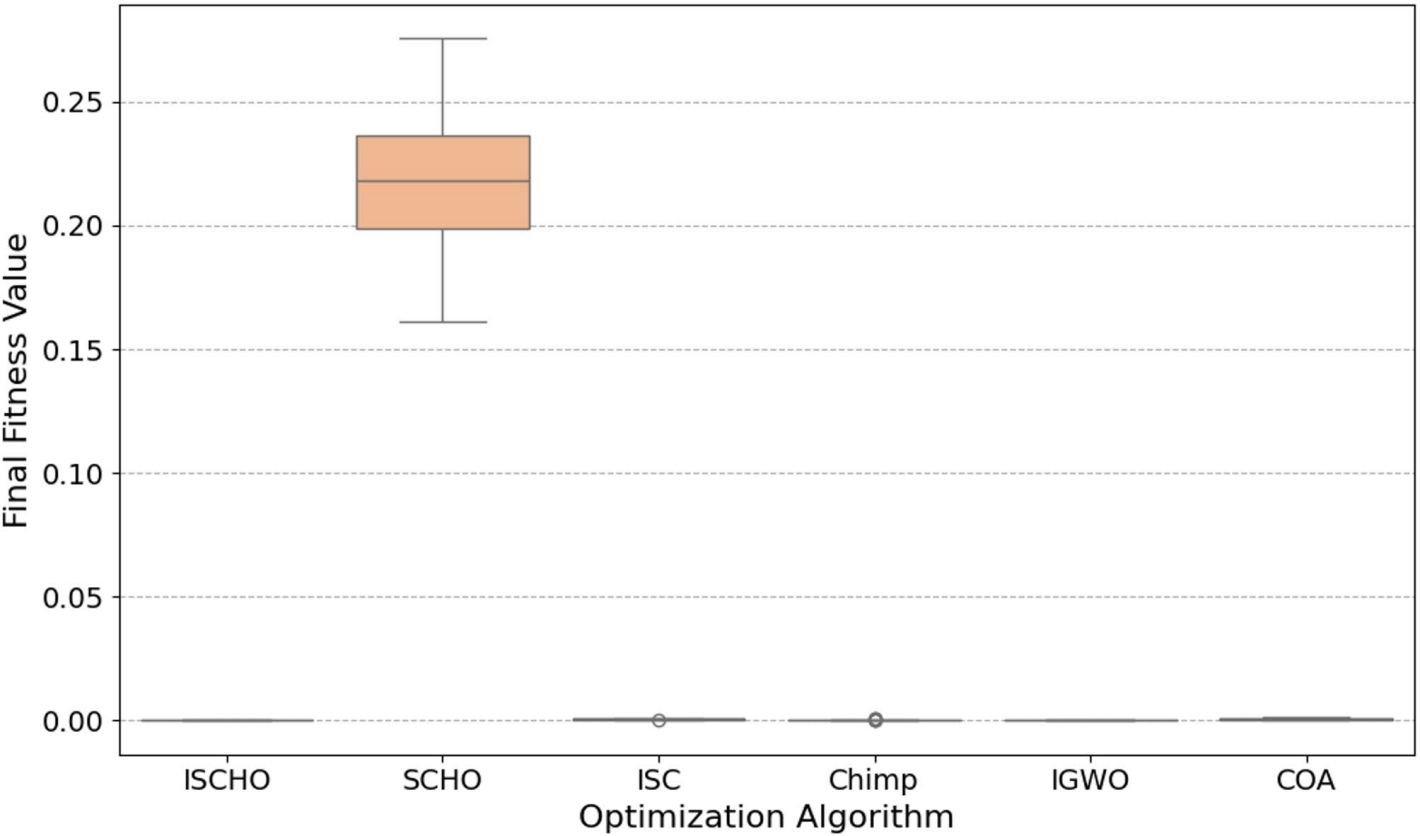
The Boxplot of the cost function across independent runs using Max_t_ = 1000 and *n* = 500.

**Fig. 15:**
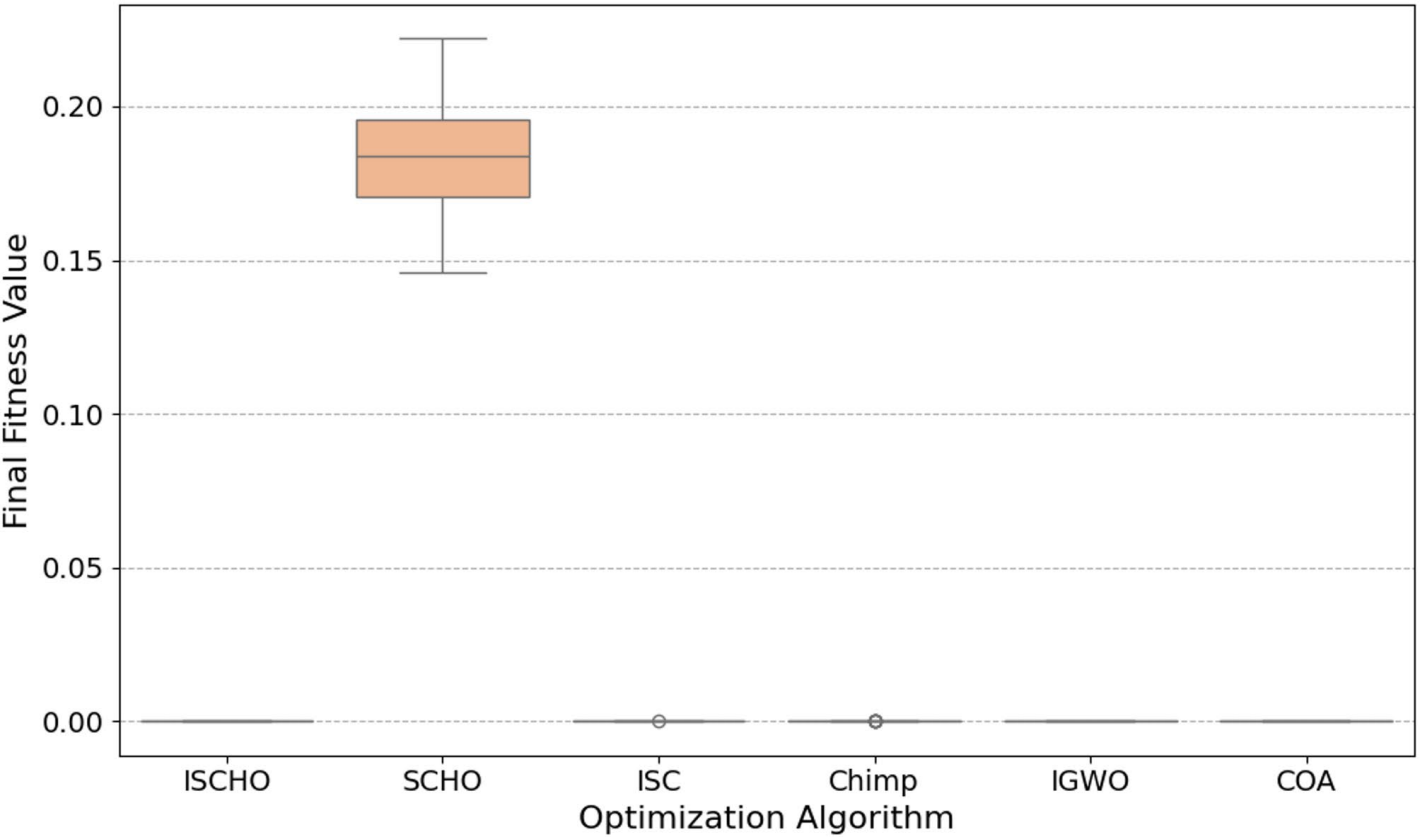
The Boxplot of the cost function across independent runs using Max_t_ = 1000 and *n* = 1000.

In this section, ISCHO will be tested and compared to other optimizers using n= (500 and 1000) at Max_t_ =1000. Figures 12 and 13 represent the fitness values of all optimizers according to each iteration number using *n* = 500 and 1000, respectively. Figures 14 and 15 represent the boxplot of the fitness (cost) function across independent runs (iterations) for the proposed ISCHO against other algorithms using Max_t_ =1000 and n= (500 and 1000).Table 8 includes a comparison between optimizers at n= (500 and 1000) and Max_t_ =1000 using F, ET (second unit), Mean, and Std. measurements.

It is noted that the F and Mean values for all optimizers presented in Table 8 (at Max_t_=1000) are better than their values provided in Table 6 (at Max_t_=200) and Table 7 (at Max_t_=500) according to n equals 500 and 1000.

**Table 8:** A comparison between optimizers at n= (500 and 1000) and Max_t_ = 1000.

Algorithm	Metrics							
	*n* = 500				*n* = 1000			
	F*10^− 6^	ET	Mean*10^− 6^	Std.	F*10^− 6^	ET	Mean*10^− 6^	Std.
ISCHO	0	4.16298	0	0	0	7.90156	0	0
SCHO	7.9705	4.942664	7.9705	0	1.1503	10.76538	1.1503	0
ISC	2.9293	15.632916	2.9293	0	1.4987	31.56963	1.4987	0
Chimp	0.59532	4.186178	0.59532	0	0.46283	15.53336	0.46283	0
IGWO	0.93248*10^− 8^	37.559377	0.93248*10^− 8^	0	0.16956*10^− 16^	117.3601	0.16956*10^− 16^	0
COA	0.01*10^− 20^	6.3599259	0.01*10^− 20^	0	0.05*10^− 29^	14.66874	0.05*10^− 29^	0

On the other hand, the execution time is increased for all optimizers when Max_t_ is increased. Thus, the execution time of all optimizers at Max_t_=1000 is more than their execution time at Max_t_=200 and Max_t_=500. The results in Figs. 12 and 14, and Table 8 illustrate that ISCHO outperformed SCHO, ISC, Chimp, IGWO, and COA at n equals 500 because it provided zero value for F, Mean, and Std. Thus, the first best optimizer is ISCHO, the second best optimizer is COA, and the third best optimizer is IGWO. On the other hand, the worst optimizer is SCHO because it provided the maximum F and Mean (F = 7.9705*10^− 6^, Mean = 7.9705*10^− 6^).

As presented in Figs. 13 and 15, and Table 8, it is proved that ISCHO is superior to other optimizers at n equals 1000. Thus, the first best optimizer is ISCHO, the second best one is COA, and the third best one is IGWO. On the other hand, the worst optimizer that provided the maximum F and Mean values is ISC. In all cases, Std. value is zero for all optimizers at both population sizes (*n* = 500 and *n* = 1000). According to ET values at n equal to 500 and 1000, the maximum execution time is provided by IGWO while the minimum execution time is provided by ISCHO. Thus, at Max_t_ =1000, ISCHO can provide the best results at the minimum execution time compared to other optimizers. At the end, ISCHO can be used as the best energy management system that can provide the optimal energy value according to each energy source.

In fact, ISCHO is essentially proposed to address the challenge of achieving efficient energy generation for renewable energy management while minimizing operational costs, a critical aspect where traditional optimization methods often fall short due to the complex trade-offs involved. To overcome this, the ISCHO is a new meta-heuristic algorithm provided to enhance energy management systems in Nano-grids. ISCHO works by optimizing key parameters related to many energy sources (PV, Wind, generators, and batteries), all with the explicit goal of minimizing the total operational cost. The energy management process is framed as an optimization problem where the cost function is critically dependent on the generated energy from each source and its current price. This design allows ISCHO to dynamically adjust energy distribution parameters; consequently, if the price of energy from any source changes, the optimization algorithm will recalculate and provide an updated, optimized energy management strategy based on the new price. Our simulation results and comparative analysis consistently demonstrate that ISCHO achieves a lower total cost in Nano-grid management compared to traditional optimization methods, thereby proving its superior performance in real-time scenarios and effectively navigating the compromise between efficiency and cost.

### Statistical evaluation of ISCHO comparing to the other algorithms

This section details the statistical evaluation of the proposed ISCHO algorithm against the other five algorithms listed in Table 2, which also specifies their parameter settings during execution. The comparison will be performed at a maximum of 1000 iterations (Max_t_ = 1000) and with a population size of 1000 search agents. Five statistical methods will be employed: Mean, Std., CI, *t*-test (with a significance level of α = 0.05), and Wilcoxon test (with a p-value threshold of *p* ≥ 0.05)^32,33^. These analyses will be conducted using the five benchmark functions detailed in Table 4. The results of the Mean, Std., and CI measurements, used to assess ISCHO’s performance relative to the other five algorithms according to these benchmark functions, are presented in Table 9a and b. Additionally, Table 10 provides the outcomes of the *t*-test and Wilcoxon test, which also serve to compare ISCHO against the other five algorithms using the same five benchmark functions. At the end, the performance of ISCHO to demonstrate its generalizability and robustness will be tested using k-fold cross-validation (k = 10) based on two different energy management datasets called KU-HMG dataset^31^ and IEEE microgrid test cases dataset^34,35^.

**Table 9:** Statistical analysis of ISCHO via others using mean, std., and CI.

(a)									
Function	Algorithm								
	Chimp			IGWO			COA		
	Mean	Std.	CI	Mean	Std.	CI	Mean	Std.	CI
F1	0.189	0.0012	[0.185, 0.193]	0.0965	0.0008	[0.096, 0.097]	0.1253	0.0011	[0.1251, 0.1255]
F2	0.169	0.0012	[0.163, 0.172]	0.1536	0.0012	[0.1533, 0.1539]	0.0956	0.00079	[0.0955, 0.0957]
F3	**− 0.985**	**0.004**	**[− 1003**,** − 0.99]**	3.2656	0.0295	[3.265, 3.2662]	3.985	0.0298	[3.9848, 3.9852]
F4	3.56	0.029	[3.5, 3.62]	0.2454	0.00185	[0.245, 0.2458]	0.4698	0.0033	[0.4696, 0.47]
F5	0.689	0.0053	[0.686, 0.692]	0.0687	0.0005	[0.0685, 0.0689]	0.0535	0.00043	[0.0515, 0.0555]

(b)									
Function	Algorithm								
	ISCHO			SCHO			ISC		
	Mean	Std.	CI	Mean	Std.	CI	Mean	Std.	CI
F1	0.007	0.000066	[0.0067, 0.0073]	0.078	0.0006	[0.075, 0.081]	0.165	0.00014	[0.163, 0.169]
F2	0.006	0.000053	[0.0056, 0.0064]	0.145	0.0014	[0.142, 0.148]	0.12	0.0011	[0.1, 0.14]
F3	3.0008	0.02505	[3.00078, 3.00028]	3.245	0.035	[3.241, 3.249]	3.458	0.025	[3.454, 3.462]
F4	0.04	0.00036	[0.02, 0.06]	1.005	0.0125	[1.001, 1.009]	1.345	0.0124	[1.34, 1.35]
F5	0.008	0.00007	[0.0078, 0.0082]	0.078	0.0006	[0.075, 0.081]	0.645	0.00535	[0.64, 0.65]

Significant values are in bold.

**Table 10:** Statistical analysis of ISCHO via others using *t-*test and *p-*value.

Function	Algorithm									
	SCHO		ISC		Chimp		IGWO		COA	
	t-test	*p*-value	t-test	*p*-value	t-test	*p*-value	t-test	*p*-value	t-test	*p*-value
F1	0.011	3.02 × 10^–12^	0.002	3.00 × 10^–10^	0.302	3.03 × 10^− 7^	0.005	3.00 × 10^− 9^	0.011	3.06 × 10^–10^
F2	0.009	3.05 × 10^− 2^	0.009	5.02 × 10^− 4^	0.001	3.02 × 10^− 4^	0.002	3.00 × 10^− 3^	0.01	3.05 × 10^− 5^
F3	0.01	3.02 × 10^− 3^	0.006	3.32 × 10^− 4^	0.011	2.02 × 10^− 4^	0.002	3.32 × 10^− 2^	0.011	3.02 × 10^− 5^
F4	0.009	5.02 × 10^–10^	0.002	4.02 × 10^–12^	0.004	3.02 × 10^− 6^	0.011	3.12 × 10^− 1^	0.013	3.02 × 10^− 4^
F5	0.002	3.32 × 10^–12^	0.002	3.02 × 10^–10^	0.013	3.02 × 10^− 7^	0.013	3.02 × 10^–12^	0.005	4.02 × 10^–12^

Table 9a and b illustrates a statistical evaluation of the performance of several algorithms according to five functions (F1 to F5), using Mean, Std., and CI as metrics. Comparing the Mean values, ISCHO consistently gives a lower Mean for functions F1, F2, F4, and F5 than SCHO, ISC, Chimp, IGWO, and COA, indicating superior average performance.

For function F3, ISCHO has Mean value of 3.0008, which is lower than SCHO, ISC, IGWO, and COA but higher than Chimp’s -0.985. The Std. calculates the variability of the results; lower Std. values imply more consistent performance. Generally, ISCHO provides low Std. according to the benchmark functions (F1 to F2), proposing that its results are relatively consistent. The CI gives a range in which the true mean is likely to fall. In general, the CI values for ISCHO are tighter compared to other algorithms, further supporting the conclusion that ISCHO’s performance is more consistent and reliable.

The results of t-tests and p-values to compare the performance of ISCHO against other algorithms for each benchmark function are depicted in Table 10. In fact, the t-test calculates the difference between the means of two groups, while the p-value indicates the statistical significance of this difference. Hence, the p-values are consistently very small (on the order of 10^− 12^ or 10^− 10^) through all benchmark functions and all algorithm comparisons. Small p-values indicate that the differences in the means between ISCHO and the other algorithms (SCHO, ISC, Chimp, IGWO, and COA) are statistically significant. This evidence suggests that ISCHO’s performance is significantly different from the other algorithms, and given the mean values in Table 9a and b, it can be inferred that ISCHO outperforms these algorithms. Tables 11 and 12 ensure the generalizability and robustness of ISCHO according to each fold and average values using KU-HMG and IEEE microgrid test cases datasets.

**Table 11:** ISCHO’s performance at each fold based on mean and std. Metrics using KU-HMG dataset.

Fold	Mean	Std.
1	0.003070	0.000099
2	0.003071	0.000086
3	0.003071	0.000066
4	0.003072	0.000053
5	0.003072	0.000079
6	0.003073	0.000298
7	0.003073	0.000033
8	0.003073	0.000032
9	0.003073	0.000025
10	0.003073	0.000011
Average	0.00307	0.0000782

**Table 12:** ISCHO’s performance at each fold based on mean and std. Metrics using IEEE microgrid test cases dataset.

Fold	Mean	Std.
1	0.00337	0.000309
2	0.003371	0.000296
3	0.003371	0.000276
4	0.003372	0.000263
5	0.003372	0.000289
6	0.003373	0.000508
7	0.003373	0.000243
8	0.003373	0.000242
9	0.003373	0.000235
10	0.003373	0.000221
Average	0.003372	0.000288

### Sensitivity analysis

**Fig. 16:**
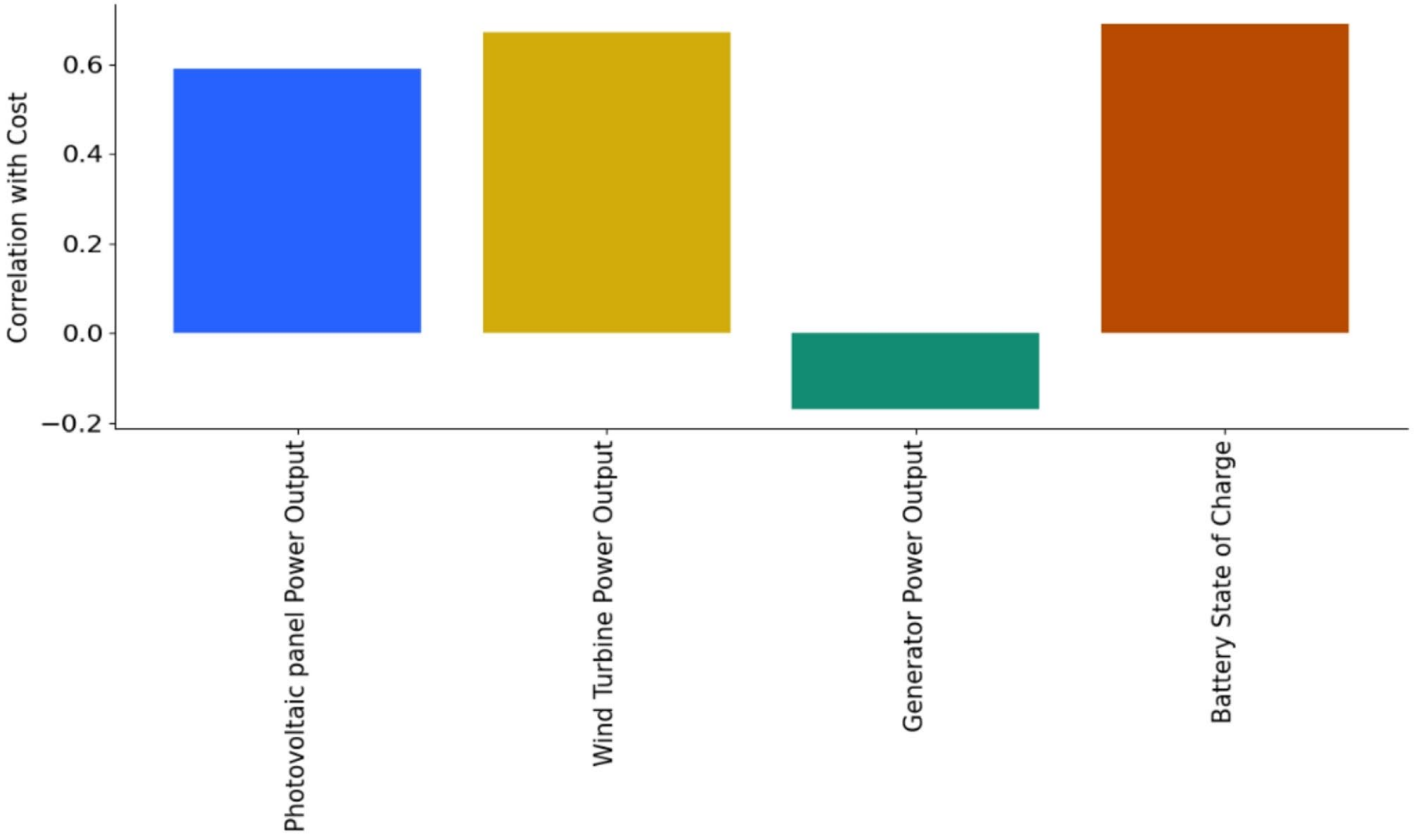
Sensitivity analysis of each parameter to the fitness (cost) function.

In this subsection, a sensitivity analysis of each parameter to the fitness (cost) function will be described. During these experiments, the values of the parameters ( Ct, u ,m,$\:\:\varvec{\epsilon\:}$, s, q, p, $\:\varvec{\alpha\:}$, and$\:\:\varvec{\beta\:}$) for the ISCHO are (3.6, 0.388, 0.45, 0.003, 0.5, 10, 9, 1.6, and 1.55) respectively, while the values of the $\:{\varvec{\varnothing\:}}_{\varvec{m}\varvec{a}\varvec{x}}$ and $\:{\varvec{\varnothing\:}}_{\varvec{m}\varvec{i}\varvec{n}}$ are 0.8 and 0.75678 as depicted in Table 2. This analysis was performed using the Pearson correlation coefficient between each parameter and the cost function, which represents the sum of these parameters. These parameters are four parameters, which are Photovoltaic panel Power Output (E_PV_), Wind Turbine Power Output (E_WIND_), Generator Power Output (E_NGG_), and Battery State of Charge (E_Bat_). The correlation coefficient measures the strength and direction of the linear relationship between two variables. A coefficient close to 0 refers to a weak or no linear relationship, a coefficient close to -1 refers to a strong negative relationship, and a coefficient close to 1 refers to a strong positive relationship. The sensitivity analysis is presented in Fig. 16; Table 13.

**Table 13:** The cost value according to each parameter.

Parameter	Cost value
Photovoltaic panel power output (E_PV_)	0.586908
Wind turbine power output (E_WIND_)	0.672466
Generator power output (E_NGG_)	− 0.179648
Battery state of charge (E_Bat_)	0.690722

In Fig. 16; Table 13, E_PV_ exhibits a moderate positive correlation (0.587), implying that solar panel output affects the cost function, though less strongly than E_Bat_ or E_Wind_. Accordingly, higher E_PV_ corresponds to a moderate increase in cost value. E_Wind_ has a substantial positive correlation (0.672), which indicates that E_Wind_ also strongly influences the cost. Hence, increased E_Wind_ is associated with a notable rise in cost. E_NGG_ illustrates a weak negative correlation (-0.180), referring to a minimal inverse relationship with the cost. Increases in E_NGG_ have a slight tendency to decrease the cost, but this effect is not very pronounced. E_Bat_ displays the strongest positive correlation (0.691), referring to that changes in the battery’s state of charge have the most significant impact on the cost. As the E_Bat_ increases, the cost tends to increase noticeably. Hence, the cost function is most sensitive to changes in E_Bat_ and E_WIND_, followed by E_PV_, while it has low sensitivity to E_Bat_.

## Conclusions and future work

This study presented the Improved Sinh Cosh Optimizer (ISCHO), a novel meta-heuristic algorithm tailored for enhancing energy management in Nano-grid systems. ISCHO addresses the challenge of balancing energy efficiency with operational cost minimization across multiple distributed energy sources, including photovoltaic panels, wind turbines, natural gas generators, and battery storage. The algorithm formulates the energy management task as a constrained optimization problem, where the cost function is based on the energy output and associated operational costs of each source. ISCHO’s strength lies in its dynamic adjustment of energy distribution using a nonlinear switching mechanism between exploration and exploitation phases, ensuring robust search capability and avoidance of local minima.

Results from simulations and comparisons with various recent optimizers, including SCHO, ISC, Chimp, IGWO, and COA, were performed. Based on the KU-HMG dataset, these comparisons were conducted using particular configurations of two population sizes (500 and 1000) and three maximum iteration numbers (200, 500, and 1000). The results indicated that ISCHO had the lowest overall costs under these particular testing settings. In terms of the stated fitness value, mean, and standard deviation for this specific dataset and problem formulation, the results showed that ISCHO performed better than the other algorithms in most cases. It seemed to retain competitive execution times, especially at the maximum iteration count, while offering more optimal solutions, especially at larger population sizes and iteration counts. ISCHO showed a better ability to find lower cost solutions across the tested scenarios. Additionally, ISCHO was found to have a shorter execution time than the other optimizers at the maximum iteration number. Moreover, a sensitivity analysis based on the Pearson correlation coefficient revealed that generator output and battery charge levels had the highest impact on the cost function, reinforcing ISCHO’s effective prioritization of cost-sensitive parameters.

Despite these promising outcomes, several limitations should be acknowledged. First, the evaluation was limited to a static dataset without accounting for dynamic variations in energy demand, environmental conditions, or pricing changes, which commonly occur in real-world Nano-grid operations. Second, the model does not include battery degradation, efficiency losses, or hardware limitations, which may affect performance in practical deployments. Future research will address these limitations by incorporating time-series data, real-time constraints, and experimental validation in field-deployed energy systems to assess ISCHO’s real-world robustness and scalability. Additionally, a visual analogy for the inherent suitability of Sinh and Cosh functions for balancing act will be will be discussed in the future to enhance the paper’s accessibility for readers less familiar with these mathematical concepts.

## Data Availability

Dataset is available at: [https://data.mendeley.com/datasets/x8v796pjsx/1](https:/data.mendeley.com/datasets/x8v796pjsx/1).
